# An *in silico* analysis of robust but fragile gene regulation links enhancer length to robustness

**DOI:** 10.1371/journal.pcbi.1007497

**Published:** 2019-11-15

**Authors:** Kenneth Barr, John Reinitz, Ovidiu Radulescu

**Affiliations:** 1 Department of Genetic Medicine, University of Chicago, Chicago, Illinois, United States of America; 2 Departments of Statistics, Ecology & Evolution, Molecular Genetics & Cell Biology, University of Chicago, Chicago, Illinois, United States of America; 3 LPHI UMR CNRS 5235, University of Montpellier, Montpellier, France; Ottawa University, CANADA

## Abstract

Organisms must ensure that expression of genes is directed to the appropriate tissues at the correct times, while simultaneously ensuring that these gene regulatory systems are robust to perturbation. This idea is captured by a mathematical concept called *r*-robustness, which says that a system is robust to a perturbation in up to *r* − 1 randomly chosen parameters. *r*-robustness implies that the biological system has a small number of sensitive parameters and that this number can be used as a robustness measure. In this work we use this idea to investigate the robustness of gene regulation using a sequence level model of the *Drosophila melanogaster* gene *even-skipped*. We consider robustness with respect to mutations of the enhancer sequence and with respect to changes of the transcription factor concentrations. We find that gene regulation is *r*-robust with respect to mutations in the enhancer sequence and identify a number of sensitive nucleotides. In both natural and *in silico* predicted enhancers, the number of nucleotides that are sensitive to mutation correlates negatively with the length of the sequence, meaning that longer sequences are more robust. The exact degree of robustness obtained is dependent not only on DNA sequence, but also on the local concentration of regulatory factors. We find that gene regulation can be remarkably sensitive to changes in transcription factor concentrations at the boundaries of expression features, while it is robust to perturbation elsewhere.

## Author summary

Robustness assures that organisms can survive when faced with unpredictable environments or genetic mutations. In this work, we characterize the robustness of gene regulation using an experimentally validated model of the regulation of the *Drosophila* gene *even-skipped*. We use a mathematically precise definition of robustness that allows us to make quantitative comparisons of robustness between different genetic sequences or between different nuclei. From this analysis, we found that genetic sequences that were not previously known to be important for gene regulation reduce sensitivity to genetic perturbation. In contrast, we found that gene regulation can be very sensitive to the concentrations of regulators. This extreme sensitivity was only observed at the boundaries of expression features, where switch-like behavior is desirable. This highlights the importance of considering context when assessing robustness.

## Introduction

Biological systems must be robust to perturbations, both environmental and genetic, in order to maintain their functions in fluctuating circumstances [1]. Some of their robustness properties, such as noise reduction, are shared with general cybernetic systems. Others, such as the relationship between robustness and evolvability are specific to living systems. Robustness of general cybernetic systems was studied mathematically by von Neumann [2]. von Neumann employed multiplexing and majority rule with Boolean automata, an approach that captured buffering by redundancy of the error control, but failed to treat flexibility and the possibility of adaptation underlying biological robustness. Many authors tried to describe the peculiarities of biological robustness using metaphors such as robust-yet-fragile [3], René’s Thom’s theory of catastrophes [4–6] or control theory [7]. Although, like von Neumann’s automata, biological organisms are remarkably robust to uncertainty in their components, they can be strongly influenced by small perturbations that act on sensitive elements of regulatory control networks [1]. As shown by large scale multiple knock-out analysis of metabolic networks in yeast, regulatory networks are robust with respect to single gene mutations, but become sensitive when multiple genes are mutated [8]. It has also been shown using mathematical modeling that segmentation patterns in the *Drosophila* embryo are robust to changes of single parameters [9]. Similarly, the accuracy of the gap gene response to a maternal gradient is unaffected by mutations of a single gene [10], but is, as predicted by theory, sensitive to a double mutation [11, 12]. Furthermore, the relation between robustness, redundancy and complexity is particularly relevant to biological systems. It has been suggested, that, contrary to the common belief that simple systems are robust, a certain degree of complexity can also lead to stable behavior [13, 14]. This property follows from the very general mathematical principle of measure concentration in high dimension [15, 16], as first discussed in Gorban and Radulescu [13]. The law of large numbers is an instance of this principle, ensuring that non-correlated variation of additive effects is buffered and vanishes when the number of elements increases. Within the same formalism, the concept “robust yet fragile” is made precise by the idea of *r*-robustness: a functional property can be stable with respect to perturbation of up to *r* − 1 randomly chosen parameters and sensitive when *r* parameters are varied simultaneously [13]. Robust biological systems include organismal development, where a form of robustness called canalization assures that all individuals arrive at the same phenotype despite individual genetic variation [17–19]. Genetic and signal transduction network models have provided mechanistic explanations of developmental robustness and canalization in *Drosophila* [11, 12], *C. elegans* [20], and *S. purpuratus* [21].

In gene networks, the connections between genes represent regulatory interactions which control levels of expression of genes through *cis*-regulatory elements, typically 500 to 1000 basepairs (bp) in length, called enhancers [22]. These sequences contain clusters [23, 24] of binding sites for transcription factors (TFs) that act in combination to direct gene expression in specific spatial domains or tissues. While it is well understood how the dynamics of developmental networks confer robustness to the system, it is poorly understood how or if the enhancers that control these networks contribute to robustness through their organization. An important property of enhancers is their redundancy, which is seen at two levels. The clusters of binding sites in an enhancer typically include multiple binding sites for the same TF, conferring a many-to-one relationship between TF binding sites and gene expression [25]. At a higher level, multiple enhancers can control expression in the same expression domain or tissue type [26], and such “shadow” enhancers are known to increase robustness [27–32]. These specific experimental findings have remained largely unaddressed at the theoretical level.

Previously described data driven and experimentally well tested models of *Drosophila* development are an ideal system for the theoretical study of robustness. Confocal microscopy has been used to generate spatial and temporal atlases of protein and mRNA levels at single nucleus resolution during the first 4 hours of development [33–38]. These data provide the basis of sequence level models of gene regulation, which predict gene expression levels as a function of protein levels and DNA sequence [39–48]. Using such models, it is possible to address how the general principles of gene regulation can confer robustness to mutations in enhancers.

In this work we use a previously reported model of gene regulation [47] to model the robustness of the *Drosophila even-skipped* (*eve*) locus with respect to variation in both TF concentration and DNA sequence. This model is described fully in the Appendix to this work and its main features are listed in the Results section. *eve* codes for the homeodomain protein Eve, whose expression forms seven sharply located stripes, necessary for the formation of parasegments during embryonic development [49]. We specifically assess two types of robustness: distributed robustness and *r*-robustness [13]. The robustness of this gene regulation model has never been investigated. The previously introduced robustness concepts have only been tested on a signalling network model and never in developmental biology. We find that the regulation of *eve* can be extraordinarily sensitive to certain changes in TF concentrations, a property that may help form sharp borders in expression domains. We also find that this regulation is *r*-robust with respect to sequence mutation. Expression of *eve* is sensitive only to changes in a few nucleotides of the enhancer. Finally, we show that the number of sensitive nucleotides decreases in longer enhancer sequences from both natural and *in silico* generated enhancers, indicating that enhancer length confers robustness to genetic perturbation. We thus provide a computational proof of the importance of enhancer length for the robustness of the gene regulation.

## Results

### Distinguishing types of robustness

Distributed and *r*-robustness arise in complex systems whose properties depend on a large number of parameters. In the former case, the effect of a single perturbation is small and grows very slowly with the number and size of perturbations. In the latter, weaker case, the system is insensitive to the majority of perturbations, excepting the perturbation of a few sensitive parameters. Gorban and Radulescu [13] formalized these types of robustness and investigated the robustness of a well described signaling pathway. In this work we follow the definitions laid out in Gorban and Radulescu [13, Eqs 1 and 2]. We consider the robustness of a positive quantitative property *M* that depends on *n* positive parameters *K* = (*K*_1_, *K*_2_, ⋯, *K*_*n*_), namely *M* = *f*(*K*_1_, *K*_2_, ⋯, *K*_*n*_). The property *M* is robust *in a distributed manner* with respect to changes in these parameters if the variance in *M* is reduced compared to the variance in the parameters *K*, when the parameters are subjected to independent perturbations. That is, considering variance in all independent parameters Var(log *K*_*i*_) = Var(log *K*), *i* ∈ {1, ⋯, *n*}, then we consider *M* to be robust in a distributed manner if

$$\begin{array}{c} \text{Var}(\text{log}\;M)\ll \text{Var}(\text{log}\;K). \end{array}$$ (1)

Similarly, if we consider a subset of *r* parameters *I*_*r*_ = {*i*_1_, *i*_2_, ⋯, *i*_*r*_} ⊂ 2^{1,⋯,*n*}^, which we multiply by positive, independent, identically distributed, random scales (*s*_1_, *s*_2_, ⋯, *s*_*r*_), we define *M* as *r*-robust if, there is an r* such that, for any *r* < r* and randomly chosen *I*_*r*_,

$$\begin{array}{c} \text{Var}(\text{log}\;M)\ll \text{Var}(\text{log}\;s), \end{array}$$ (2)

where Var(log *s*) is the variance of each log *s*_*i*_, 1 ≤ *i* ≤ *r*. According to this definition, an *r*-robust property *M* can have large variance if the number of perturbed parameters is larger or equal to r*.

To distinguish between these types of robustness, it is useful to study the relationship between the variance of parameters and the variance in the output, or similarly to observe the variance in the output given the number of parameters perturbed.

For example, consider a system and a property *M* that is *r*-robust. In this system, there are *n* parameters, of which *n*_0_ parameters are individually sensitive to perturbation, meaning that *M* is sensibly affected by the perturbation of each of these parameters. For a formal definition of sensitivity with respect to individiual parameters one can use either the ratio Var(log *M*)/Var(log *K*_*i*_) or the usual local sensitivity measure $|\frac{\partial \;\text{log}\;M}{\partial \;\text{log}\;K_{i}}|$ averaged over the domain of interest in the parameter space. If we select *r* of *n* parameters at random, the probability we did not select a sensitive parameter is (1 − *n*_0_/*n*)^*r*^. Then the probability that at least one sensitive parameter was selected is 1 − (1 − *n*_0_/*n*)^*r*^. If changes in a sensitive parameter contribute *V*_0_ to the log-variance, and the effect is not cumulative, then the log-variance in *M* with respect to *r* mutations is given by

$$\begin{array}{c} \text{Var}\left(\text{log}\;M\right)=\left(1-{\left(1-n_{0}/n\right)}^{r}\right)V_{0}. \end{array}$$ (3)

Although *V*_0_ is large, Var(log *M*) is small for small *r* and becomes *V*_0_ only when *r* is large enough. The cross-over value of *r* characterizing the loss of robustness is smaller for a large number of sensitive parameters *n*_0_.

The definition of *n*_0_ uses two implicit assumptions: i) that parameters have a well defined structural or biochemical meaning (nucleotide, concentrations of transcription factors, binding affinities, etc.) and ii) that among the identified parameters some are highly sensitive and the others have negligible sensitivity. These two assumptions are not always satisfied. For instance, the total number of parameters and the number of sensitive parameters can be different in models with different levels of abstraction. As sensitive parameters are related hierarchically across levels of abstraction we expect that findings for one type of model apply to other models in a hierarchy of abstractions (a full discussion of hierarchies of models related by model reduction can be found elsewhere [50]). Rather generally, we expect that a number of parameters have sensitivity much higher than the others. This phenomenon was explained from first principles in models with time scales distributed over many orders of magnitude. As discussed elsewhere [13], the heterogeneity of the sensitivity of parameters of biochemical systems results from the existence of widely distributed time and concentration scales, a property called “multiscaleness.” In multiscale systems some parameters are important and dominate the others, while a majority of parameters have small effect and play a more static role. The term “sloppy-sensitivity” is sometimes used to designate this situation. However, in practice, a threshold must be chosen to separate sensitive from non-sensitive parameters. This threshold can be for instance chosen inside the largest gap in the distribution of sensitivities. Interestingly, the relation (3) does not need knowledge of the threshold to identify the number of sensitive parameters *n*_0_. It is enough to perform a *r*-robustness test and the value of *n*_0_ is found by fitting Eq (3). Furthermore, let us define *r*_1/2_ as the number of simultaneously perturbed parameters producing a variance Var(*M*) that is half of the maximal variance *V*_0_. It follows straightforwardy from (3) that

$$\begin{array}{c} n_{0}=n\left(1-2^{-\frac{1}{r_{1/2}}}\right). \end{array}$$ (4)

Eq (4) implies that *n*_0_ and *r*_1/2_ are negatively correlated, the decrease of the first and the increase of the latter equivalently meaning higher *r*-robustness.

To summarize, the term *r*-robustness means nonlinear dependence of the variance on the number *r* of randomly chosen perturbed targets according to a saturation curve described by Eq (3). *r*_1/2_ is the characteristic value of *r* separating robust (low variance) and non-robust (high variance) situations. In models relating DNA sequence to gene expression each nucleotide in the sequence is a parameter and point mutations act on a single parameter, thus the number of sensitive parameters represents the number of sensitive nucleotides.

In contrast to the *r*-robustness situation, consider the function *M* = (*K*_1_*K*_2_ ⋯ *K*_*n*_)^1/*n*^. This function has distributed robustness. In this case, if all parameters have a log-variance of *V*_*K*_, then the log-variance of *M* with respect to *r* will be

$$\begin{array}{c} \text{Var}(\text{log}\;M)=\frac{V_{K}r}{n^{2}}, \end{array}$$ (5)

where *r* is the number of parameters that have been independently perturbed. In contrast to *r*-robustness, the signature of distributed robustness is a linear increase of the variance with respect to *r*. Another signature of distributed robustness is the dependence of the variance on the total number of parameters when all the parameters are perturbed. If all parameters are perturbed, the log-variance of *M* is simply

$$\begin{array}{c} \text{Var}\left(\text{log}\;M\right)=V_{K}/n, \end{array}$$ (6)

and the log-variance is a vanishingly small fraction of *V*_*K*_ when *n* → ∞. While the log-variance of the geometric mean scales like 1/*n*, some other robust functions have faster variance decrease with *n*. General distributed robustness is related to concentration of measure in high-dimensional spaces, a phenomenon well known in mathematics. The distribution of a function *f* of *n* variables “concentrates,” meaning that it has vanishing variance when *n* is very large if the function depends on all of the variables but not particularly strongly on some of them (i.e., the function should have the Lipshitz property, discussed elsewhere [13]).

In the cases of *r*-robustness or distributed robustness we can assign a number that describes the robustness of the system. Rather generally, robustness is described by the ratio of variance of the input to variance of the output, a parameter we call *ρ*. For much of this work we will be working with variables of unknown scale. In light of this, we observe the variance in the fold-change of the input and the fold-change of the output. The fold-change variations are well captured by the variance of the logarithm. More precisely,

$$\begin{array}{c} \text{Var}\left(\text{log}\left(K\right)\right)=\text{Var}\left(\text{log}\left(s_{K}\right)\right),\;\text{Var}\left(\text{log}\left(M\right)\right)=\text{Var}\left(\text{log}\left(s_{M}\right)\right), \end{array}$$ (7)

where *s*_*K*_ = *K*/*K*′, *s*_*M*_ = *M*/*M*′ are the fold changes of *K* and *M* with respect to the reference values *K*′ and *M*′, respectively. Thus, the robustness ratio *ρ* is given by

$$\begin{array}{c} \rho =\frac{\text{Var}(\text{log}(M))}{\text{Var}(\text{log}(K))}. \end{array}$$ (8)

For *r*-robust properties we expect that *ρ* depends on the number *r* of perturbed targets according to

$$\begin{array}{c} \rho \left(r\right)=1-{\left(1-n_{0}/n\right)}^{r}, \end{array}$$ (9)

where *n*_0_ is the number of sensitive parameters. Thus *ρ* is small only for small *r* and a more appropriate measure of robustness is in this case *n*_0_.

### Robustness of mRNA levels with respect to transcription factor concentration

**Fig 1:**
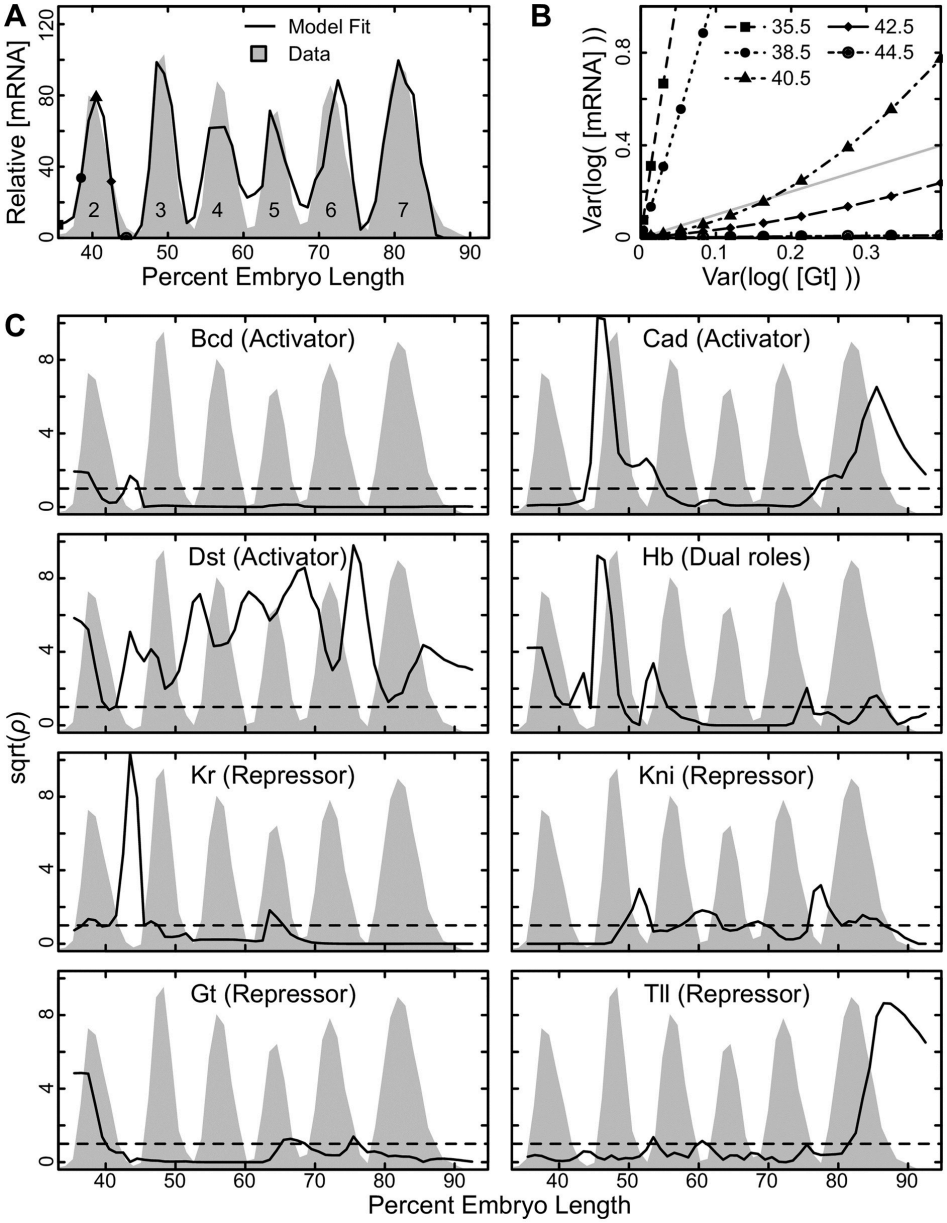
Robustness of *eve* expression to variation in TF concentration. (A) The relative expression of *eve* mRNA along a 10% wide strip along the anterior posterior axis (gray shading) and the model fit to the same data (black line). *eve* stripe number is indicated. (B) The relationship between variation in fold-change TF concentration and fold-change mRNA levels (Eq (7)) for the TF Gt at the percent embryo length indicated. The line representing *ρ* = 1 (Eq (8)) is indicated with a gray line. Points below this line are robust, while points above are sensitive. (C) The ratio of the variance in fold-change mRNA to the variance of fold change TF concentration *ρ* (Eq (8)) for the indicated TF at each position in the embryo (solid line). The role of each factor (activator or repressor) is indicated in parentheses. *ρ* values have been square root transformed for better visual presentation. The dashed line indicates the value *ρ* = 1. The perturbation size *A* in Eq (10) was set to 0.1. The expression pattern of *eve* (gray shading) is included for visual orientation.

Enhancers interpret the local concentration of transcription factors in order to specify appropriate production of mRNA. In order to determine the degree to which known regulatory mechanisms acting on enhancers contribute to robustness with respect to fluctuations in TF concentrations, we utilized a model that simulates the regulation of *Drosophila* *eve* [47], which is expressed in seven transverse stripes across developing *Drosophila* embryos. This model incorporates several mechanisms. The binding of TFs to DNA, including the effects of steric competition and cooperativity, is treated by thermodynamics [51]. Because chromatin state is an important predictor of TF binding [52–55], we exclude TF binding within closed chromatin. Other mechanisms, described phenomenologically, are short-range quenching of transcriptional activators [56–58], and coactivation of repressors [44, 59, 60]. The functional roles of the TFs used in the model are known from independent experiments, and expression is calculated by summing the bound activators after accounting for the effects of the mechanisms listed above and passing the resulting net activation *N* through a diffusion limited Arrhenius rate law, taking into account competition for interaction with the basal transcriptional machinery. In the study cited above, the model is able to accurately treat the expression pattern of stripes between 35.5% and 92.5% embryo length (Fig 1A), identify the enhancers within the *eve* regulatory locus, and simulate the effect of ectopic Hb expression. A detailed description of the model is provided in the Supplementary information, S1 Text.

To test how this system responds to fluctuations in TF levels, we simulated changes in TF levels by multiplying them by the fold ratio

$$\begin{array}{c} s_{T}=\exp\left(AX\right), \end{array}$$ (10)

where *A* is a parameter that sets the size of fluctuations, and *X* is a random number drawn from a uniform distribution between -1 and 1. Because TF levels are in arbitrary units, we observe the fold change in TF levels and the fold change in resulting mRNA and computed the ratio *ρ* (Eq (8)) after simulating 10,000 fluctuations.

We find that sensitivity to fluctuations in individual TFs varies with respect to position in the embryo. For instance, if we observe sensitivity to fluctuations in the TF Giant (Gt) at the interstripes, borders, and peak of the second *eve* stripe (positions indicated in Fig 1A), we find that at the anterior interstripe and border, expression is not robust to changes in Gt regardless of the magnitude of fluctuation (Fig 1B).

This embodies the well established fact that Gt controls the anterior border of *eve* stripe 2 [25, 61, 62]. In contrast, the posterior interstripe of stripe 2 is insensitive to fluctuations in Gt. Notably, at the peak of stripe 2, expression is robust against small fluctuations and sensitive to large ones (Fig 1B).

In general, we note that expression at interstripes is more sensitive to fluctuating TF levels than expression at stripe peaks (Fig 1C, S1 Fig). We note sensitivity to Hb at the anterior borders of stripes 3 and 4 and the posterior border of stripe 6. The posterior border of stripe 2 is sensitive to Kr. The posterior borders of stripes 3 and 4 and anterior borders of stripes 6 and 7 are sensitive to Kni. The anterior border of stripe 2 and the posterior border of stripe 5 are sensitive to Gt. Finally, the posterior border of stripe 7 is sensitive to Tll. In each case, the border is sensitive to the factors that set the border of that stripe within the embryo [25, 61–67].

### The *eve* locus is *r*-robust with respect to nucleotide changes

**Fig 2:**
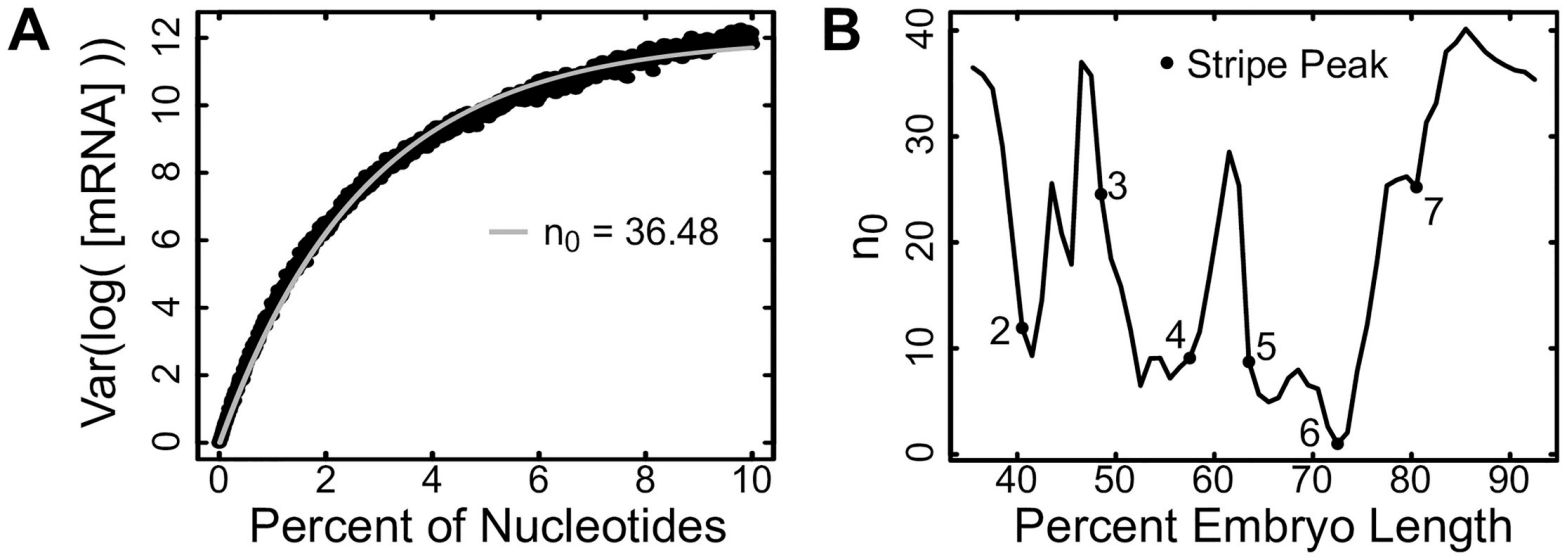
Robustness of *eve* expression to mutation of DNA sequence. (A) The variation of *eve* expression at 35.5% embryo length at various values of *r* nucleotides that are mutated. The fit to data (Materials and Methods) is shown as a grey line, and the estimated number of sensitive nucleotides, *n*_0_, is indicated (Eq (3)). (B) The number of sensitive nucleotides, *n*_0_ at every position along the anterior-posterior axis.

Genetic systems may also be robust with respect to changes in DNA sequence. In order to investigate the robustness of the *eve* locus with respect to sequence perturbation, we simulated random mutations to *r* nucleotides 10,000 times, with *r* spanning 1 to 10% of all nucleotides (see Materials and Methods Section 5.3). If *eve* expression is robust in a distributed manner, variance will increase linearly with *r* (Eq (5)). In contrast, if *eve* expression is *r*-robust, variance will saturate with increasing *r* (Eq (3)). When we examine the relationship between variance in *eve* expression and *r* (illustrated at 35.5% embryo length in (Fig 2A), we find that variance saturates with *r*, along a curve well described by Eq (3). According to the first part of the Results section, this shows that *eve* expression is *r*-robust with respect to nucleotide changes. For *r*-robust systems, robustness is captured by the parameter *n*_0_ (Eq (3)), which indicates the number of sensitive parameters. To find *n*_0_, we fit Eq (3) at every position along the anterior-posterior axis. We find that stripe peaks are more robust to perturbation than interstripes in that they have fewer sensitive parameters (Fig 2B).

### Longer *eve* enhancers are more robust to perturbation

**Fig 3:**
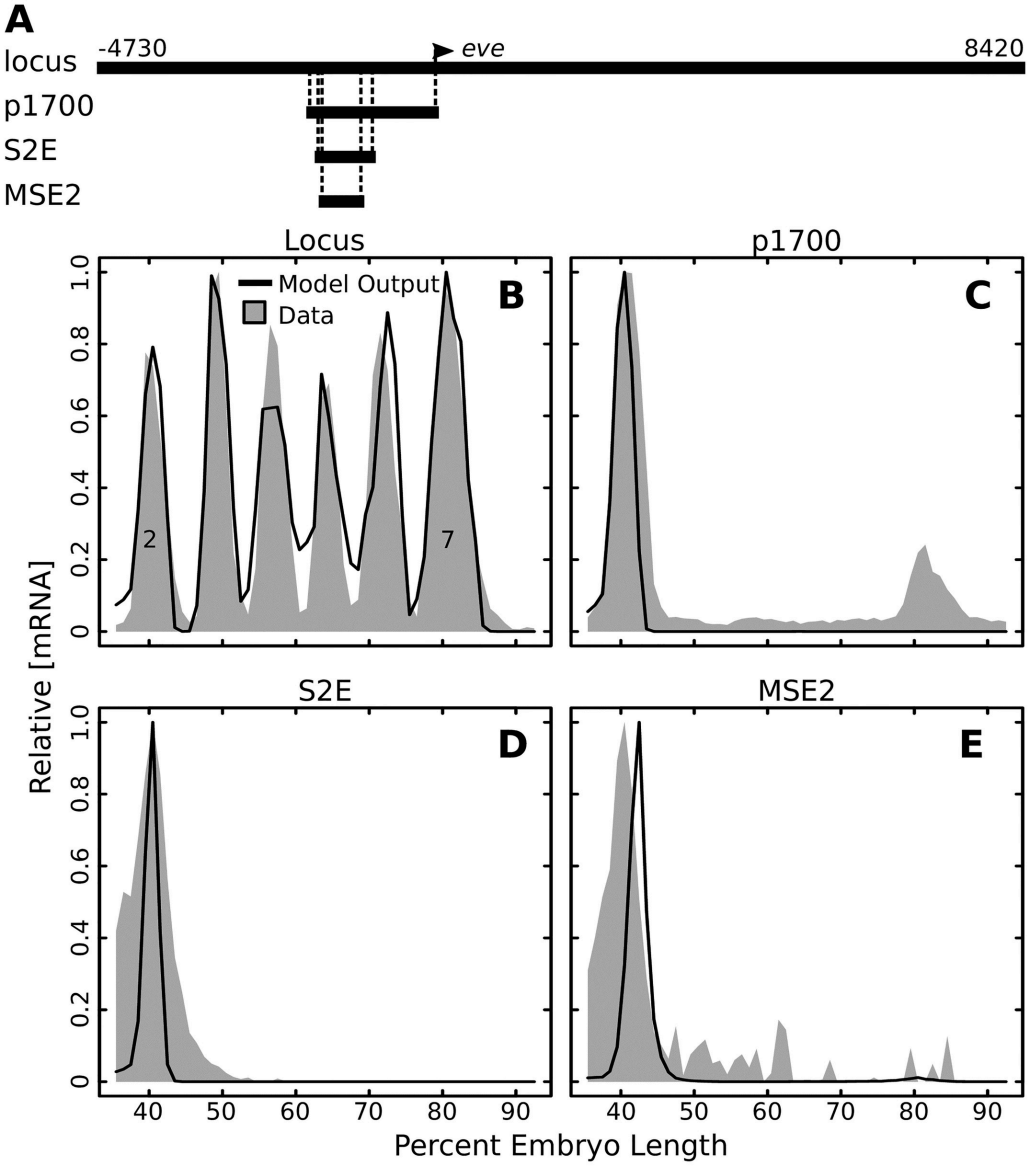
Predicted expression driven by successively smaller enhancers. (A) Diagram showing the entire *eve* locus and successively smaller sequences that all drive stripe 2. Where each sequence aligns within the locus is indicated with dashed lines. Position with respect to TSS is indicated. (B-E) The model predicted expression and actual expression for each of the sequences in (A) along the anterior-posterior axis. Model output is in black lines and expression data is in gray shading. Relative expression on a 0 to 1 scale is reported.

**Fig 4:**
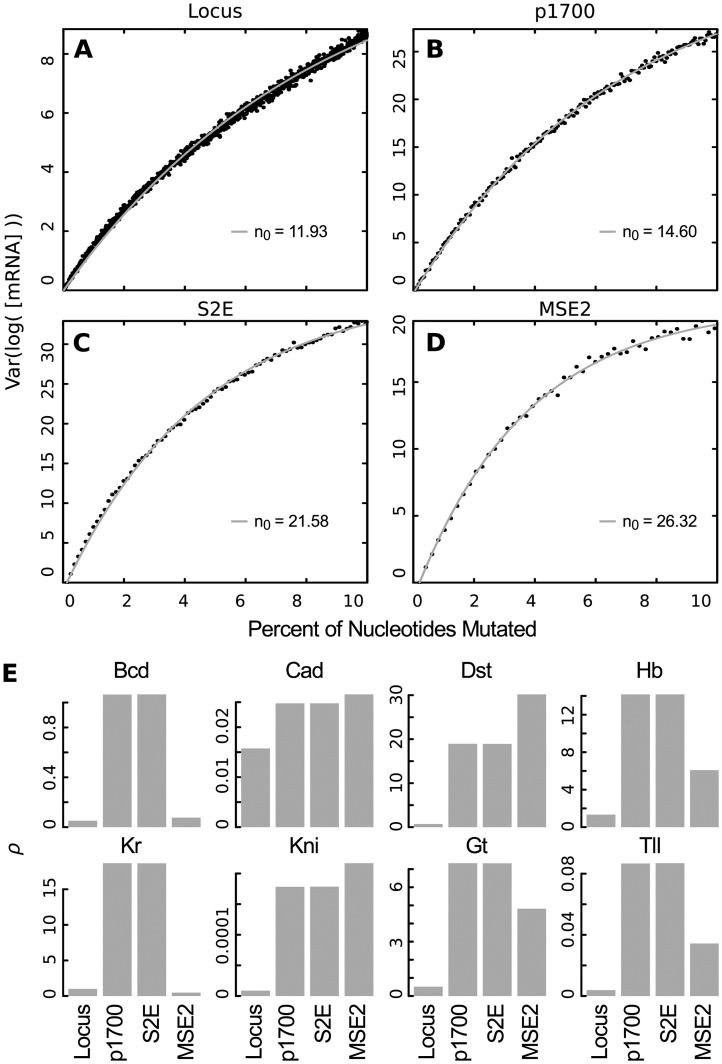
Mutational robustness of natural S2Es. (A-D) The variation in mRNA expression for increasing numbers of perturbed nucleotides at 40.5% embryo length (peak of stripe 2) is shown for sequences of different length that drive *eve* stripe 2. The best fit curve and estimated number of sensitive nucleotides *n*_0_ is indicated. (E) The ratio of variation in mRNA to variation in TF concentration *ρ* (Eq (8)) at 40.5% embryo length for each TF and each sequence. *A* was set to *A* = 0.1 in Eq (10) for simulations.

For the second stripe of *eve*, four sequences of different length are known to drive expression: the intact locus, the proximal 1700 bp, S2E, and MSE2. Each larger sequence contains the sequence of all smaller enhancers (Fig 3A). The gene regulatory model successfully predicts that each of these sequences drives expression at the position of *eve* stripe 2. The model prediction and experimental data for each enhancer is shown in Fig 3B–3E. In order to investigate whether additional sequence contributes additional robustness, we investigated the number of sensitive nucleotides *n*_0_ in each of these four sequences at the peak of stripe 2 expression (40.5% embryo length). We find that as sequence length grows, not only does the ratio of sensitive nucleotides decrease, the absolute number of sensitive nucleotides decreases from about 26 to 12 (Fig 4A–4D).

We also investigated the robustness (*ρ*) to changes in TF concentration for each of the stripe 2 enhancers. For the majority of TFs, there was a relationship between the size of the enhancer and robustness to TF concentration (Fig 4E). For the factors Cad, Dst and Kni, longer enhancers were more robust to changes in TF concentration. For Bcd, Hb, Gt and Tll MSE2 was more robust than S2E and the proximal 1700bp but less robust than the intact locus. Finally, MSE2 was the most robust to changes in Kr, followed by the intact locus, then S2E and the proximal 1700bp.

### Robustness is a function of enhancer length

**Fig 5:**
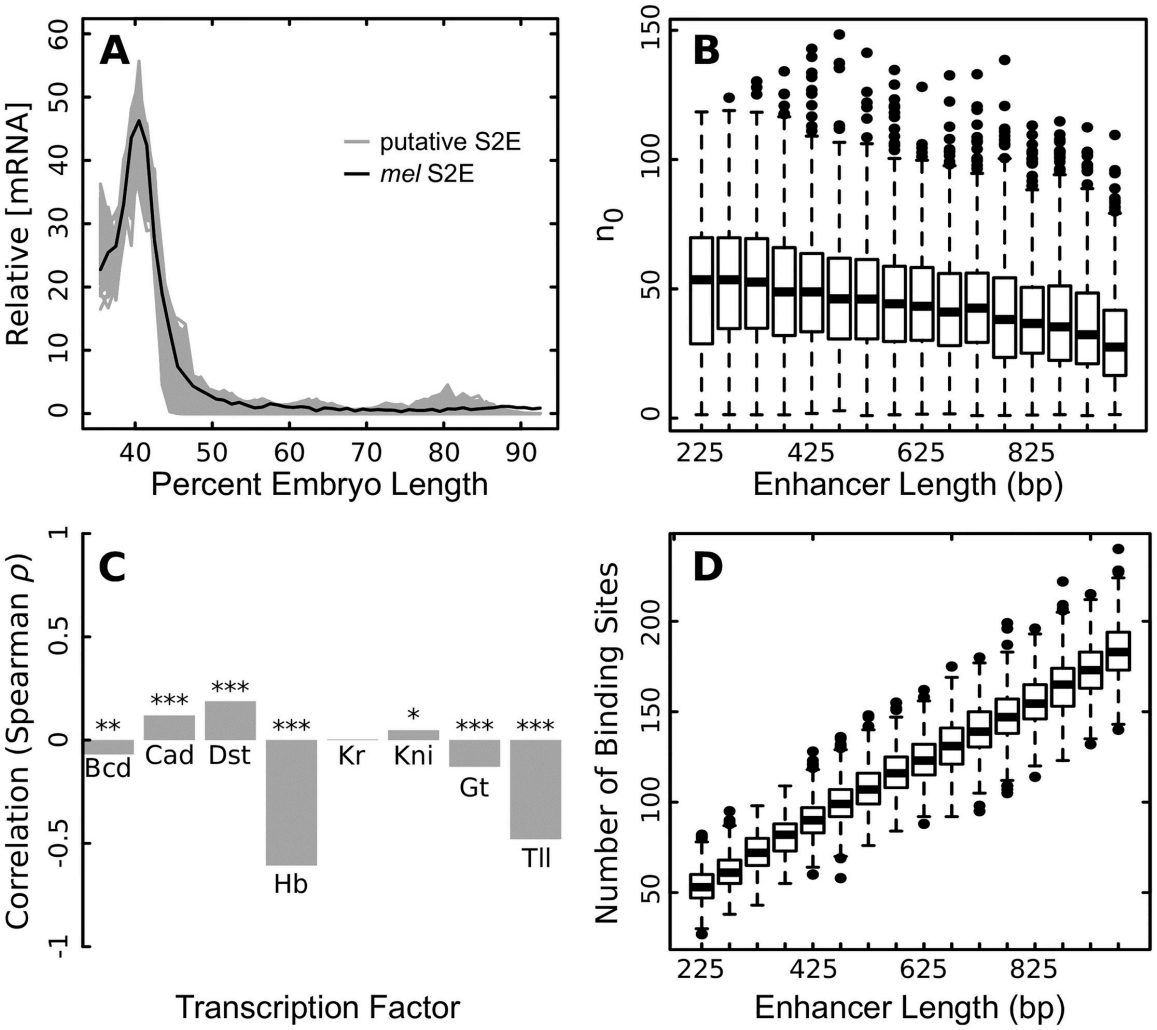
Sequence robustness of putative S2Es. (A) Predicted expression of 8010 putative S2Es with lengths from 200 bp to 1000 bp (10 each, gray lines). Each putative S2E is predicted to drive expression similar to the natural *D. melanogaster* S2E. (B) Boxplots of the number of sensitive nucleotides *n*_0_ *vs*. sequence length for each of the 8010 putative S2Es in bins of 50 bp. *n*_0_ is significantly correlated with sequence length (Spearman *ρ* = −0.25, *p* < 2.2 × 10^−16^). (C) The correlation (Spearman *ρ*) between the sensitivity to changes in transcription factor concentration (*ρ*) and the length of putative S2Es (* *p* < 10^−4^; ** *p* < 10^−9^; *** *p* < 10^−16^). The ratio of the variance in mRNA to the variance in TF levels, *ρ* (Eq (8)) was estimated with *A* = 0.1 at 40.5% embryo length. (D) Boxplots of the number of modeled binding sites in each putative S2E *vs*. sequence length for each of the 8010 putative S2Es in 50 bp bins. Spearman *ρ* = 0.96, *p* < 2.2 × 10^−16^.

In order to determine whether the relationship between sequence length and the robustness measure *n*_0_ to mutation is inherent to the system, we generated 8010 putative stripe 2 elements *in silico*. We generated 10 putative S2Es with each length from 200 bp to 1000 bp. All 8010 S2Es are predicted to drive the correct expression pattern (Fig 5A). We estimated the number of sensitive nucleotides *n*_0_ for each of these S2Es by simulating 10,000 sets of *r* sequence mutation with *r* spanning from 1 bp to 10% of the sequence length. We estimated *n*_0_ at the peak of stripe 2 expression (40.5% embryo length). We found that *n*_0_ decreases with enhancer length (Fig 5B).

We also investigated robustness, as described by *ρ*, to changes in TF concentration for each of these 8010 enhancers. We measured *ρ* from 10,000 simulations with *A* = 0.1 at 40.5% embryo length. For most TFs, there is a relationship between enhancer length and robustness to changes in TF concentration, but the direction of this relationship was not consistent between factors (Fig 5C). As enhancers increase in length, they become more robust to changes in Bcd, Hb, Gt, and Kr and less robust to changes in Cad, Dst, and Kni.

### The location and mechanism of sensitive nucleotides

**Fig 6:**
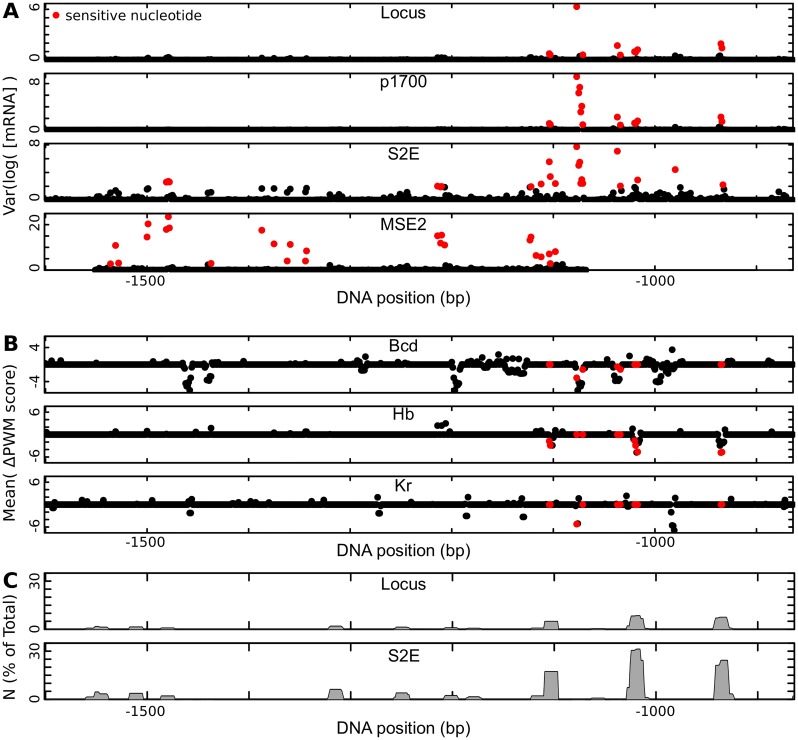
Location of sensitive nucleotides. (A) Log variance of mRNA expression when each nucleotide is perturbed one at a time, for each of the four sequences tested. The *n*_0_ most sensitive nucleotides are indicated in red. (B) The mean change in PWM score for the indicated factors when each nucleotide in S2E is perturbed. The *n*_0_ most sensitive nucleotides are indicated in red. (C) The percent of total strength of transcriptional activation *N* (see Eq. 15 S1 Text) along the DNA for the locus (top) and S2E (bottom). Sensitive nucleotides represent a smaller percent of total activation in the locus compared to S2E.

In order to identify the location of sensitive nucleotides we tested all possible single nucleotide sequence perturbations and selected the nucleotides that led to the highest log variance of mRNA expression (Fig 6A and S2 Fig). For all sequences except MSE2, the sensitive nucleotides occurred in a tight cluster at the 3’ end of the stripe 2 enhancer.

To identify which factors drive sensitivity, we tested the mean change in PWM score for the eight TFs considered here. Sensitive nucleotides tend to lead to a reduced PWM score for at least one of Bcd, Kr, and Hb, but not all binding site losses correspond to sensitive nucleotides (Fig 6B). This effect was especially strong for Hb, where sequence mutations that lead to reduced PWM scores at three Hb sites represent 6 of 11 sensitive nucleotides in the intact locus.

The degree to which any DNA sequence activates *eve* transcription is captured by the model parameter *N* (Eq. 15 S1 Text). *N* represents the sum of transcriptional activators bound to a particular DNA sequences, weighted by their strength of transcriptional activation. When we examine *N* for the enhancer S2E (Fig 6C, top), we find that a large percentage of transcriptional activation comes from three segments at the 3 prime end of S2E. These correspond to the three Hb sites that contain sensitive nucleotides (Fig 6B). These three Hb sites are responsible for a considerable amount of the total activation in S2E (Fig 6C), but the percent of total activation from these sequences is much smaller in the intact *eve* locus (Fig 6C). This distributed activation makes the *eve* locus more robust than smaller enhancers to perturbation of these sequences, which are contained in both.

### Sensitive nucleotides are more conserved

Mutations of sensitive nucleotides, which lead to a greater change in transcription rates, are likely to have a negative effect on organismal fitness. Thus, we expect sensitive nucleotides to be more conserved on average than insensitive nucleotides. To test this hypothesis, we performed an alignment of S2Es from 12 Drosophilids to assess conservation at every nucleotide. Using the sensitivity values from the intact *eve* locus we found that the sensitive nucleotides were conserved in 79.5% of species, on average, compared to a background conservation rate of 68%. The correlation between sensitivity and conservation was marginally significant (Spearman *ρ*; *p* = 0.042). This correlation supports the notion that mutations in sensitive nucleotides negatively impact fitness, but is limited by the fact that nucleotides that are not important for stripe 2 expression might be conserved for other reasons. For example, they could be important for expression of other *eve* stripes, or for expression of *eve* in additional tissues. Nucleotides may also be conserved due to mechanisms not treated by our model, such as chromatin state [52, 53]. These processes will dilute the statistical signal observed

### Human enhancers are *r*-robust with respect to nucleotide changes

Recently, Kircher *et al* [68] reported saturation mutagenesis of several human regulatory elements using a high- throughput reporter assay. The mutagenesis strategy incorporates a random number of insertions, deletions, or substitutions uniformly across the length of the enhancer. Using this data, we are able to explicitly test how variance in expression changes with the number of perturbed nucleotides. We restricted our analysis to the two enhancers that showed high reproducibility across biological replicates (IRF4 and SORT1). We found that the variation in expression saturates with increasing number of nucleotide changes, along curves well described by Eq (3), for both of these enhancers (S3 Fig).

## Discussion

In this work we assessed the robustness of enhancers with respect to changes in TF levels or sequence mutation in the context of a sequence level model of gene regulation. We found that enhancers are *r*-robust to single nucleotide sequence changes, and that this robustness increases with the length of the sequence. The precise level of *r*-robustness seen, however, is not solely dependent on DNA sequence. It depends on the state of bound TFs, and thus manifests itself experimentally as dependence on position within the embryo. Sensitivity, when observed, is coupled to biological function. This point is most clearly seen in the dependence of domain border positions on TF concentration, but is also observable in the functional role of sensitive nucleotides. We discuss each of these points below.

Our major finding is that robustness of *eve* to sequence mutation is well-described by *r*-robustness. That is, the *eve* regulatory DNA is a system with a small number of sensitive parameters. This is understandable in terms of the classic experiments that elucidated this regulation. These experiments showed that enhancer function resided in multiple binding sites for each TF, each of which could be disrupted by site-directed mutation [25, 61]. Such a picture is fully compatible with *r*-robustness with *n*_0_ equal to about 26 for MSE2, which is on the order of the number of the number of base changes required to mutate all of the binding sites for a single TF.

Our finding that *n*_0_ decreases to only 12 for the whole locus indicates that robustness increases with increasing length of regulatory DNA. This might appear to contradict the modular structure of enhancers, but it too is compatible with the experimental literature. Early experiments with enhancers sought to find the minimum fragments that could recapitulate an expression feature using non-quantitative assays [25], MSE2 is quantitatively not equivalent to the full S2E, expressing at a level 5 times lower [47]. Moreover, MSE2 provides a lower rate of rescue of lethality than does the full S2E [69]. S2E, in turn, was first identified by the presence of two conserved sequences at either end [70], but this structural feature says nothing about the actual functional limits of S2E, which are known to be larger in the closely related species *D. erecta* [71]. Moreover there is evidence from the sea squirt *Ciona* that redundancy built into enhancers ensures robust expression in appropriate tissues without disrupting specificity [72]. Redundancy also buffers environmental perturbations, which can disrupt minimal enhancers [69, 73]. We have already alluded to the existence of shadow enhancers, redundant enhancers controlling the same expression domain and thereby increasing robustness [26, 27, 29–32, 74–76].

We also compared the level of robustness of putative *in silico* stripe 2 enhancers of different lengths, and also found that longer enhancers were more robust. These results also suggest that selective forces that are not explicitly modeled here drive the evolution of robustness. These *in silico* enhancers were selected for their pattern generating capability, but not explicitly for robustness. Thus, it is interesting that both MSE2 and S2E have fewer sensitive nucleotides (26.3 and 21.6 respectively) than *in silico* enhancers of the same length (average of 49.5 and 37.1), indicating that robustness to sequence perturbation may be selected for in natural populations, a conclusion reinforced by our finding that sensitive nucleotides are better conserved across species. The high correlation between length and number of sites (Fig 5D) indicates that redundancy in binding sites combined with a more distributed contribution of all the nucleotides, with sensitive nucleotides being responsible of a lower percentage of the total activation, is probably the cause of increased robustness. Supporting this, we find that MSE2 and S2E have a higher density of sites (114 and 204) than the *in silico* enhancers of the same length (average of 100.6 and 141).

It is important to note that all these measures of sequence robustness varied according to position in the embryo. This is natural and expected because the transcriptional state is dependent not only on position, but also on bound TFs, which vary by position. As a consequence, assays for sequence and gene product concentrations provide a very incomplete picture in the absence of knowledge of state information about regulators.

In contrast with the robustness that we see in *cis-*regulatory sequence, we find marked sensitivity to TF concentrations. For example, Fig 1 shows levels of *ρ* exceeding 100. Specifically, the transcription rate at stripe borders was drastically sensitive to changes in the concentration of TFs. For instance, the anterior border of *eve* stripe 2 is controlled by Gt [25]. When Gt levels fluctuate at this embryo position, transcription levels fluctuate to a greater degree. Such sensitivity may be a necessary feature of the circuit, where high sensitivity to individual repressors allows the formation of extremely sharp borders. Fig 1 also shows areas of low sensitivity. Such areas of apparent robustness fall into two classes. First, in Fig 1C there are areas of high robustness in areas where a transcription factor is not expressed. These areas include the region posterior to 50% embryo length for Bcd, Hb between 60% and 70% embryo length, Kr posterior to 70% embryo length, Kni anterior to 45% embryo length, Gt from 45% to 60% embryo length, and Tll anterior to 80%. Here the insensitivity to perturbation arises from the trivial reason that any multiplicative factor applied to zero gives zero. Second, we see reduced sensitivity at stripe peaks. We believe this arises because of the dependence of the transcription activation on the individual transcription factors concentration is a strongly nonlinear sigmoidal function whose derivative is very large close to a threshold (domain borders) and low far away. This excludes robustness at the domain borders and allows it in between.

The results reported here are a precise and quantitative characterization of what it means for a specific biological system to be “robust but fragile.” The concept of *r*-robustness makes this idea precise, and in the case of robustness against changes of sequence, the values of *n*_0_ found have a clear relationship to well known experimental results. We see no evidence of the $1/\sqrt{n}$ concentration characteristic of the central limit theorem as illustrated in Eqs 5 and 6. This is to be expected in biological systems, where the necessity of precise control confronts the need for stability and resilience against perturbation, a concept well captured by the idea of *r*-robustness.

The *r*-robustness of *cis-*regulatory sequences is achieved by a principle of “hierarchical heterogeneity” according to which nucleotide mutations have widely distributed impacts. The total rate of transcription of a gene is a weighted sum of contributions of different DNA segments. The contribution to the total transcription of each segment is itself a sum over many interacting sites. At each level of this hierarchy, sensitive functions such as exponential binding affinities or the diffusion-limited Arrhenius rate law produce a disparity among parameters, in which sensitive parameters stand out. Sensitive nucleotides dominate the others and their impact cannot be easily flattened by addition, thus excluding distributed concentration effects. However, in longer sequences the combined effects of many nucleotides dilutes the impact of all the mutations. Therefore, some nucleotides, sensitive in short sequences become insensitive in the long ones.

## Materials and methods

### Model selection

The model used in this work is the same as reported in Barr and Reinitz [47]. The parameter set used was the best model including chromatin state information, called ‘Repeat Chromatin #2’ in that work.

### Simulations of TF concentration perturbation

We perturbed TF concentration by selecting a random number *X* distributed uniformly between -1 and 1. Then we multiplied the TF concentration at every embryonic position by exp(*AX*), where *A* is a set parameter that scales the size of perturbation, and we observed the predicted change in mRNA synthesis rate at all positions. We repeated this calculation 10,000 times for each value of *A* between 0 and 3 in increments of 0.1, for a total of 310,000 simulations. This was repeated for each of the 8 TFs included in the model.

### Simulations of sequence mutation

In the intact locus model, some nucleotides are not accessible for TF binding because of the chromatin state. We perturbed DNA sequence by selecting sets of *r* nucleotides only from open chromatin regions. The *r* nucleotides were then substituted by one of the remaining three possible nucleotides with equal probability. We then assessed model output at each position. This was repeated for every set *r* from 1 10% of the total accessible nucleotides.

### Estimation of sensitive nucleotides

To fit Eq (3) to data, we used simulated annealing from the R package GenSA, using default parameters. *n* was set to the total length of accessible nucleotides, or 8765 for the locus, 1726 for p1700, 804 for S2E and 484 for MSE2.

### Enhancer-reporter assays

The locus expression, as well as the expression of the S2E and MSE2 construct are reported in Barr and Reinitz [47]. The p1700 data is from Janssens *et al*. [39].

### Generation of putative S2Es

To generate putative S2Es of different lengths, we fixed the kinetic parameters and optimized DNA sequence using previously described methods [77]. We used the expression of S2E as the target. We started each optimization with a random sequence of the desired final length.

### 12 species alignment

To identify conservation at sensitive nucleotides we first obtained putative S2E sequences by using the BLAST tool at FlyBase [78]. We identified significant contiguous alignments for the species *sim, sec, ere, yak, rho, ele, tak, eug, bia, kik*, and *pse*. We performed an alignment using Clustal Omega [79] using default parameters. To get a conservation score at every base in the *melanogaster* sequence, we calculated the percent of species containing the same nucleotide as *melanogaster* at that position.

### Analysis of human enhancers

Data from Kircher *et al*. [68] was obtained from the NCBI Gene Expression Omnibus under accession number GSE126550. We restricted our analysis to enhancers of SORT1 and IRF4, which had high reproducibility across biological replicates. This was necessary because our analysis of variance would otherwise be dominated by variance due to measurement error. For every barcode, we counted the number of unique molecular identifiers (UMIs) observed in the DNA and RNA in each of three experimental replicates. Additionally, for each barcode we counted the number of mutations that had been introduced to the original sequence. We excluded mutations that were not observed in the DNA pool in all any of the experimental replicates. The ratio of UMI counts in RNA to UMI counts in DNA serves as a measure of expression. We examined the variance in log-expression for each number *r* perturbed nucleotides. We reported this number for all values of *r* that represent at least 100 barcodes. When we fit the values of 3 we also include a linear adjustment for experimental measurement error.

## Supporting information

S1 Text Sequence level model of the gene regulation. A text describing the sequence level model of gene regulation is provided.
(PDF)

S1 Fig Heatmap of robustness of *eve* expression to variation in TF concentration. A heatmap comparing the variance in fold-change input to fold-change output (Eqs 7 and 8) Var(log(Δ[mRNA])) at different positions within the embryo as well as different sizes of perturbation to TF concentration, indicated by Var(log(Δ[TF])). Darker shading represents increasing variation in mRNA levels.
(TIF)

S2 Fig **Sensitive nucleotides change with** *r* (Top) The log variance in S2E mRNA expression when each nucleotide is perturbed one at a time (*r* = 1). The top 26 most sensitive nucleotides are indicated in red. (Bottom) The log variance in S2E mRNA expression when each nucleotide is perturbed in a pairwise fashion with all other nucleotides (*r* = 2). The 26 most sensitive nucleotides from the *r* = 1 are indicated in red.
(TIF)

S3 Fig *r*-robustness in human enhancers. The log variance in expression with respect to the number of mutated nucleotides is reported for each of three experimental replicates for enhancers for the human genes SORT1 and IRF4. The best fit to Eq (3) (red) and the corresponding number of sensitive nucleotides *n*_0_ is shown. For details on this analysis see Materials and Methods.
(TIF)
