# Supplementary material for: An in silico analysis of robust but fragile gene regulation links enhancer length to robustness
Source: PLoS Comput Biol. 2019 Nov 15;15(11):e1007497. doi: 10.1371/journal.pcbi.1007497 (PMC6881076; doi:10.1371/journal.pcbi.1007497)
Supplement: S1 Text — A text describing the sequence level model of gene regulation is provided. (PDF) [file pcbi.1007497.s001.pdf]

S1 Text : sequence level model of the gene regulation

Kenneth Barr[1*], John Reinitz[2], Ovidiu Radulescu[3**],

1 Dept. of Genetic Medicine, University of Chicago, Chicago, IL, USA
2 Depts. of Statistics, Ecology & Evolution, Molecular Genetics & Cell Biology, University of Chicago, Chicago, IL, USA
3 LPHI UMR CNRS 5235, University of Montpellier, Montpellier, France

* kenneth.a.barr@gmail.com
** ovidiu.radulescu@umontpellier.fr

Summary of the model

The sequence level model of gene regulation used in this work was the subject of prior investigation [1]. This calculation has two major sections. In the first, chemical rules are used to define the equilibrium binding of transcription factors (TFs) on DNA. In the second, phenomenological rules—which restate important experimental results in the form of carefully selected equations—are used to compute the effects of DNA-bound transcription factors on local gene expression. The full calculation includes six steps: (1) calculation of TF binding affinity to DNA using position weight matrices (PWMs), (2) calculation of fractional occupancy at each binding site identified in step 1, (3) coactivation of Hunchback by locally bound Bicoid or Caudal, (4) quenching of activators by locally bound repressors, (5) weighted summation of unquenched activators along DNA, and (6) transcriptional activation though a diffusion limited Arrhenius rate law. In the sections that follow, we introduce the equations for each of these steps.

PWM scores

First, we calculate PWM scores along DNA. We index binding sites with index i and TFs with index a. We represent the binding site i for TF a, spanning nucleotides m through n using the notation $i[m, n; a]$. When it is necessary to discuss the positioning and possible overlap of binding sites, we denote by m_i, n_i, a_i the leftmost and the rightmost positions of nucleotides (measured on the DNA in the $5'$ to $3'$ direction) spanning the binding site i, and the TF binding the site i, respectively. The binding site indices i are ordered such that $m_i < m_j$ for any $i < j$. If $n_i \geq m_j$ the sites i, j, $i < j$ overlap and are competing.

The PWM score S for each binding site i of the TF a is given by

$$S_{i[m,n;a]} = \sum_{k=m}^{n} \ln\left(\frac{P_a(k-m, j_k)}{P_{\mathrm{bg}}(j_k)}\right) \tag{1}$$

where $j_k \in \{A, C, T, G\}$ is the nucleotide observed at position k, $P_a(k-m, j)$ is the probability of observing this nucleotide at position $k-m$ in a binding site for factor a, and $P_{\mathrm{bg}}(j_k)$ is the probability of observing this nucleotide in the null distribution, which we take to be the *D. melanogaster* nucleotide frequencies: $P_{\mathrm{bg}}(C) = P_{\mathrm{bg}}(G) = 0.203$; $P_{\mathrm{bg}}(A) = P_{\mathrm{bg}}(T) = 0.297$. We only keep binding sites with positive scores, representing sites that are more likely to be binding sites than the background distribution.

The PWM score S is used to calculate the binding affinity K [2].

$$K_{i[m,n;a]} = K_a^{\max} \exp\left(\frac{S_i - S_a^{\max}}{\lambda_a}\right) \tag{2}$$

where $S_a^{\max}$ is the maximum score possible for the PWM of factor a and λ_a is a positive proportionality constant.

Adjustment for non-specific binding affinity

Binding affinity has both sequence specific and non-specific contributions. We only want to consider specifically-bound TFs in our calculations. Previous work has shown that non-specific binding energy is approximately three orders of magnitude smaller than the maximum specific binding energy [3]. Following the derivation described [1], we calculate an effective binding affinity,

$$K_{i[m,n;a]}^{\mathrm{ef}} = \frac{K_{i[m,n;a]}}{1 + K_a^{\mathrm{ns}}[\mathrm{TF}_a]} \tag{3}$$

where K^{ns} is the non-specific binding energy of TF a, which we fix to $0.001 K_a^{\max}$.

Fractional Occupancy

The fractional occupancy represents the portion of time any particular binding site i is bound by transcription factor a. This can be computed using from the transcription factor concentration and the binding affinity given in Eq. (3). To calculate fractional occupancy, we add the Boltzmann weight of all binding states that contain a bound transcription factor, divided by the sum of the Boltzmann weights of all the binding states, known as the partition function. We take into account the cooperativity of binding of close sites and the competition for binding between overlapping sites.

To this aim we introduce $q_i, 0 \leq q_i$, representing the Boltzmann weight of the bound state of the site i. The weight q_i is proportional to the product between the effective binding affinity and the concentration of the transcription factor, giving

$$q_i = K_{i[m,n;a]}^{\mathrm{ef}}[TF_a]/([TF_a]^{\max} K_a^{\max}). \tag{4}$$

We also define a function that specifies the index of the rightmost (closest to $5'$) non-competing binding site to the left (in the direction towards $5'$) of the site i:

$$f\big(i[m,n;a]\big) = \max_{n_k \leq m_i, k<i} \big(k[m,n;a]\big). \tag{5}$$

If there is no non-competing binding site left of the site i, then $f\big(i[m,n;a]\big) = 0$.

We define iteratively the following three partial partition functions:

$$\begin{aligned} Z_i &= Z_{i-1} + q_i Z_{f(i)} + \sum_{k=1}^{i-1} q_i q_k w(i,k) Z_{f(k)},\ Z_0 = 1 && (6) \\ Z_i^{\mathrm{c}} &= \sum_{k=1}^{i-1} q_i q_k w(i,k) Z_{f(k)}, && (7) \\ Z_i^{\mathrm{nc}} &= q_i Z_{f(i)}, && (8) \end{aligned}$$

where $w(i,k)$, with $0 \leq w(i,k) \leq 1$, is the cooperative interaction strength between sites i and k. $w(i,k)$ is only nonzero for Bcd sites less than 60 bp apart.

We compute the partial partition functions while scanning DNA in the direct (5' to 3') and in the reverse (3' to 5') directions. We denote these Z^+,Z_i^{c+}, Z_i^{nc+}, and Z^-,Z_i^{c-}, Z_i^{nc-}, respectively. From these quantities, we compute the fractional occupancy as

$$f_{i[m,n;a]} = \frac{Z_i^{nc+} Z_i^{c-} + Z_i^{c+} Z_i^{nc-} + Z_i^{nc+} Z_i^{nc-}}{Z_n q_i}, \tag{9}$$

where $n = \max i$. A more detailed description is given elsewhere [4].

Coactivation

For sites i and k, the distance between sites is computed as

$$d(i,k) = \min(|m_i - n_k|, |n_i - m_k|), \tag{10}$$

and the efficiency of interaction is given by

$$g(i,k;A,B) = \begin{cases} 1 & d(i,k) \leq A \\ 1 - \frac{d(i,k)-A}{B} & A < d(i,k) < A+B \\ 0 & A+B \leq d(i,k) \end{cases}, \tag{11}$$

where A and B are positive parameters governing the shape of this interaction.

We divide fractional occupancy into two states: an activating state f^A and a quenching state f^Q. For obligate repressors $f^Q_{i[m,n;a]} = f_{i[m,n;a]}$, and similarly for obligate activators $f^A_{i[m,n;a]} = f_{i[m,n;a]}$. For factors that coactivate,

$$f^Q_{i[m_i,n_i;a_i]} = f_{i[m_i,n_i;a_i]} \prod_{k \neq i} \left(1 - g(i,k;D_c,50) E^C_{a_k} f_{k[m_k,n_k;a_k]}\right), \tag{12}$$

$$f_i^A = f_i - f_i^Q \tag{13}$$

where D_c is a free parameter giving the maximum distance at which coactivation is 100% efficient and $E^C_{a_k}$, $0 \leq E^C_{a_k} \leq 1$, is a free parameter giving the maximum efficiency with which the factor a_k induces activation of factor a_i. This product occurs over all k binding sites within the locus.

Quenching

The effective occupancy of each activator, F, is given by

$$F_i = f_i^A \prod_{k \neq i} \left(1 - g(i,k,100,50) E^Q_{a_k} f_k^Q\right), \tag{14}$$

where $E^Q_{a_k}$, $0 \leq E^Q_{a_k} \leq 1$, is a free parameter giving the efficiency with which the factor a_k quenches. This product occurs over all k binding sites within the locus.

Summation of activating strength

The contribution to the total transcriptional activation for a sequence of bases from m to $m + \alpha$ is

$$N_{[m,m+\alpha]} = \sum_k F_k E^A_{a_k} I(k,m,m+\alpha), \tag{15}$$

where the $E^A_{a_k}$, $0 \leq E^A_{a_k} \leq 1$ is the activating efficiency of factor a_k and $I(k,m,m+\alpha)$ is a function that specifies whether the site k falls between p and q, given by

$$I(k,m,m+\alpha) = \begin{cases} 1 & m_k \geq m, n_k < m+\alpha \\ 0 & \text{Otherwise} \end{cases} \tag{16}$$

Activation by a diffusion-limited Arrhenius rate law

The rate of transcription driven by a sub-sequence bounded by m and $m + \alpha$, is given by

$$R_{[m,m+\alpha]} = \frac{R_{\max}}{1 + \exp(\theta - N_{[m,m+\alpha]})}, \tag{17}$$

where $R_{\max} \geq 0$ is the efficiency of transcription, $\theta \geq 1$ is the total energy barrier which sets the rate of transcription in the absence of activation. For a locus of length l, the fraction of time that any DNA segment $[m, m + \alpha]$ influences the promoter is given by

$$T_{[m,m+\alpha]} = \frac{\beta N_{[m,m+\alpha]}}{1 + \sum_{n=1-\alpha}^{l} \beta N_{[n,n+\alpha]}}, \tag{18}$$

where the free parameter β, $\beta \geq 0$, determines how much individual bound adaptors increase the frequency of interaction with the promoter. The total rate of transcription driven by the locus is then given by the frequency-weighted sum of transcription due to each DNA segment $[m, m + \alpha]$, so that

$$R_{\text{total}} = \sum_{m=1-\alpha}^{l} R_{[m,m+\alpha]} T_{[m,m+\alpha]}. \tag{19}$$

References

1. Barr KA, Reinitz J. A sequence level model of an intact locus predicts the location and function of nonadditive enhancers. PLoS One. 2017;12:1–26.

2. Berg G, von Hippel PH. Selection of DNA binding sites by regulatory proteins. Statistical-mechanical theory and application to operators and promoters. Journal of Molecular Biology. 1987;193:723–50.

3. Maerkl SJ, Quake SR. A systems approach to measuring the binding energy landscapes of transcription factors. Science. 2007;315:233–237.

4. Barr K. Decoding *cis*-regulation of the *Drosophila even-skipped* Locus with Physical-Chemical Models and Synthetic Enhancers. University of Chicago; 2017.
