# Supplementary figures and images for: An in silico analysis of robust but fragile gene regulation links enhancer length to robustness
Source: PLoS Comput Biol. 2019 Nov 15;15(11):e1007497. doi: 10.1371/journal.pcbi.1007497 (PMC6881076; doi:10.1371/journal.pcbi.1007497)

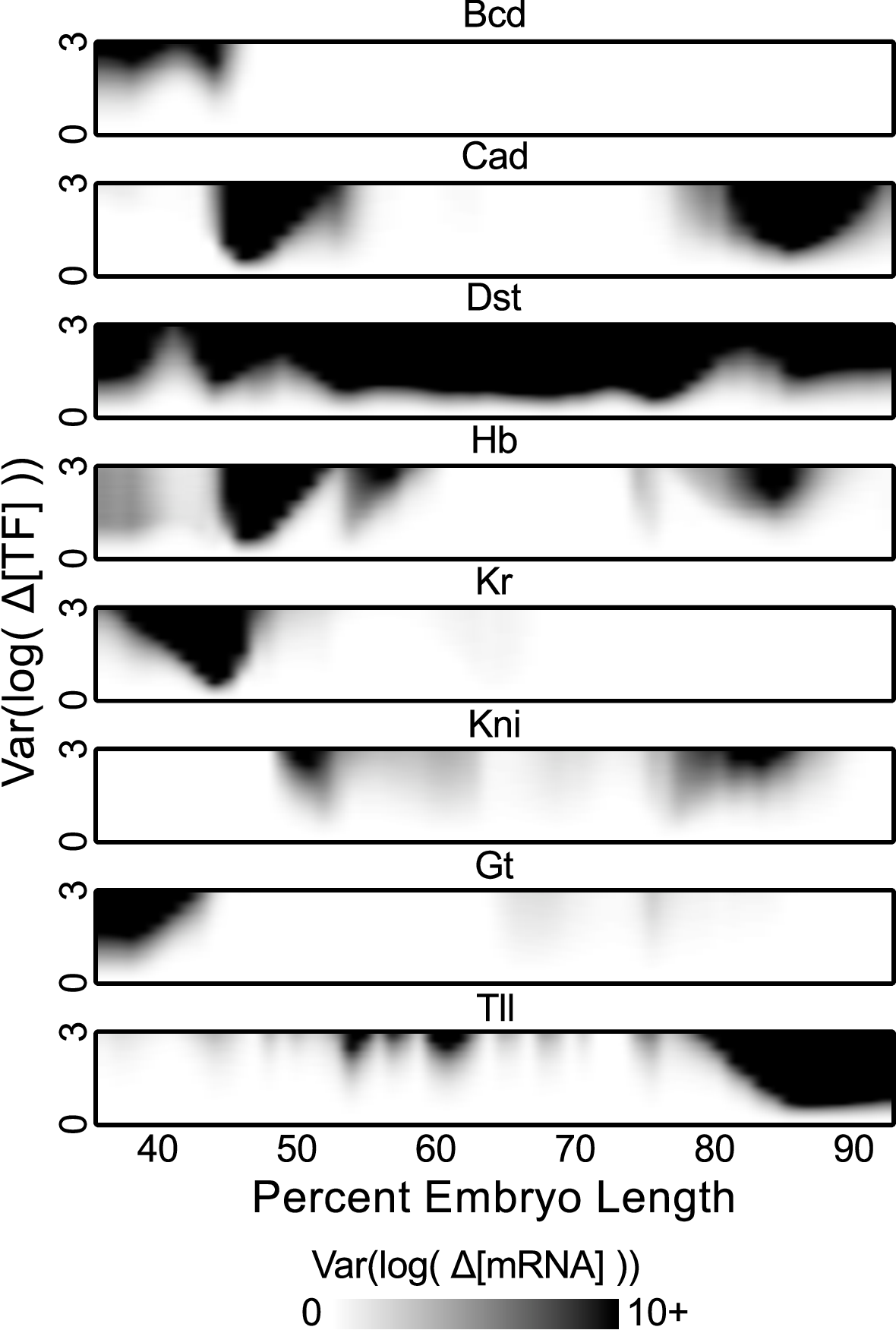

Supplement: S1 Fig — A heatmap comparing the variance in fold-change input to fold-change output (Eqs 7 and 8) Var(log(Δ[mRNA])) at different positions within the embryo as well as different sizes of perturbation to TF concentration, indicated by Var(log(Δ[TF])). Darker shading represents increasing variation in mRNA levels. (TIF) [file pcbi.1007497.s002.tif]

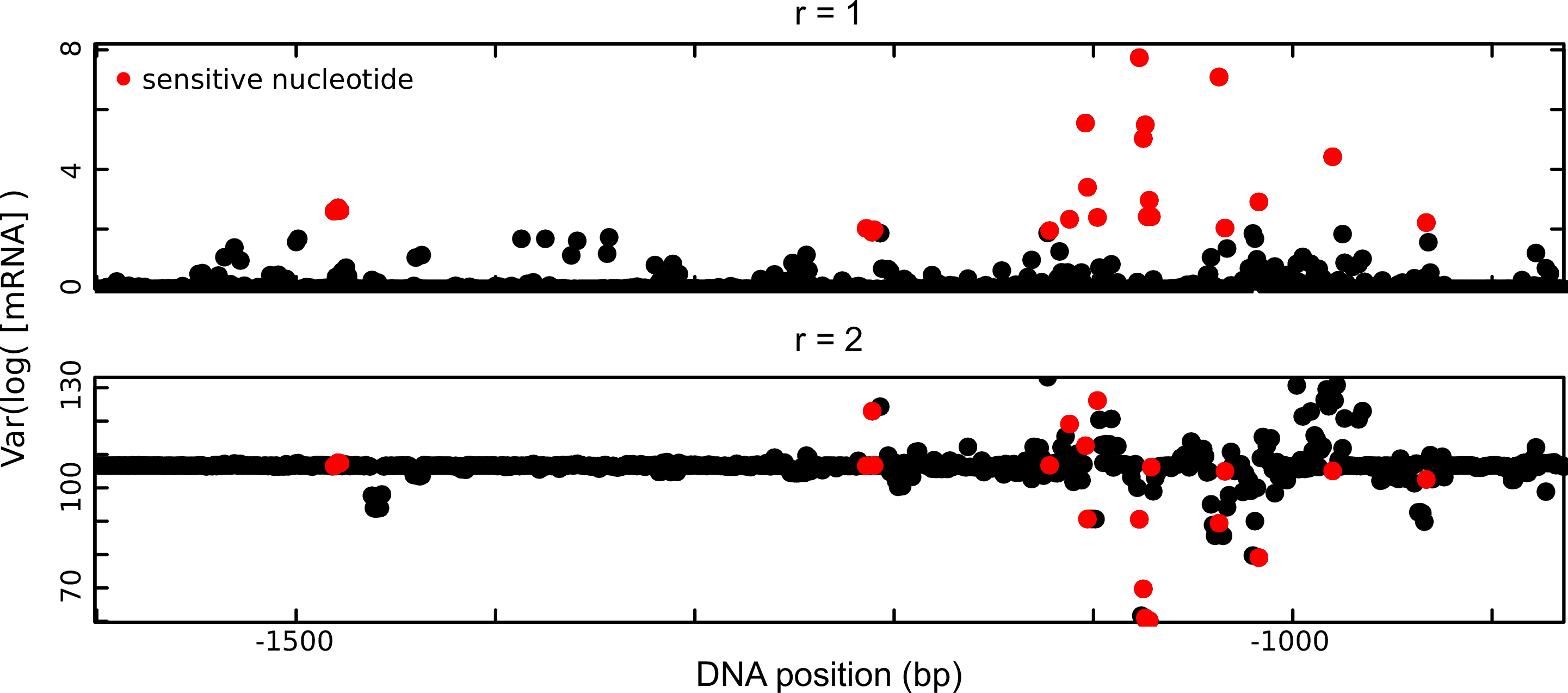

Supplement: S2 Fig — Sensitive nucleotides change with r (Top) The log variance in S2E mRNA expression when each nucleotide is perturbed one at a time (r = 1). The top 26 most sensitive nucleotides are indicated in red. (Bottom) The log variance in S2E mRNA expression when each nucleotide is perturbed in a pairwise fashion with all other nucleotides (r = 2). The 26 most sensitive nucleotides from the r = 1 are indicated in red. (TIF) [file pcbi.1007497.s003.tif]

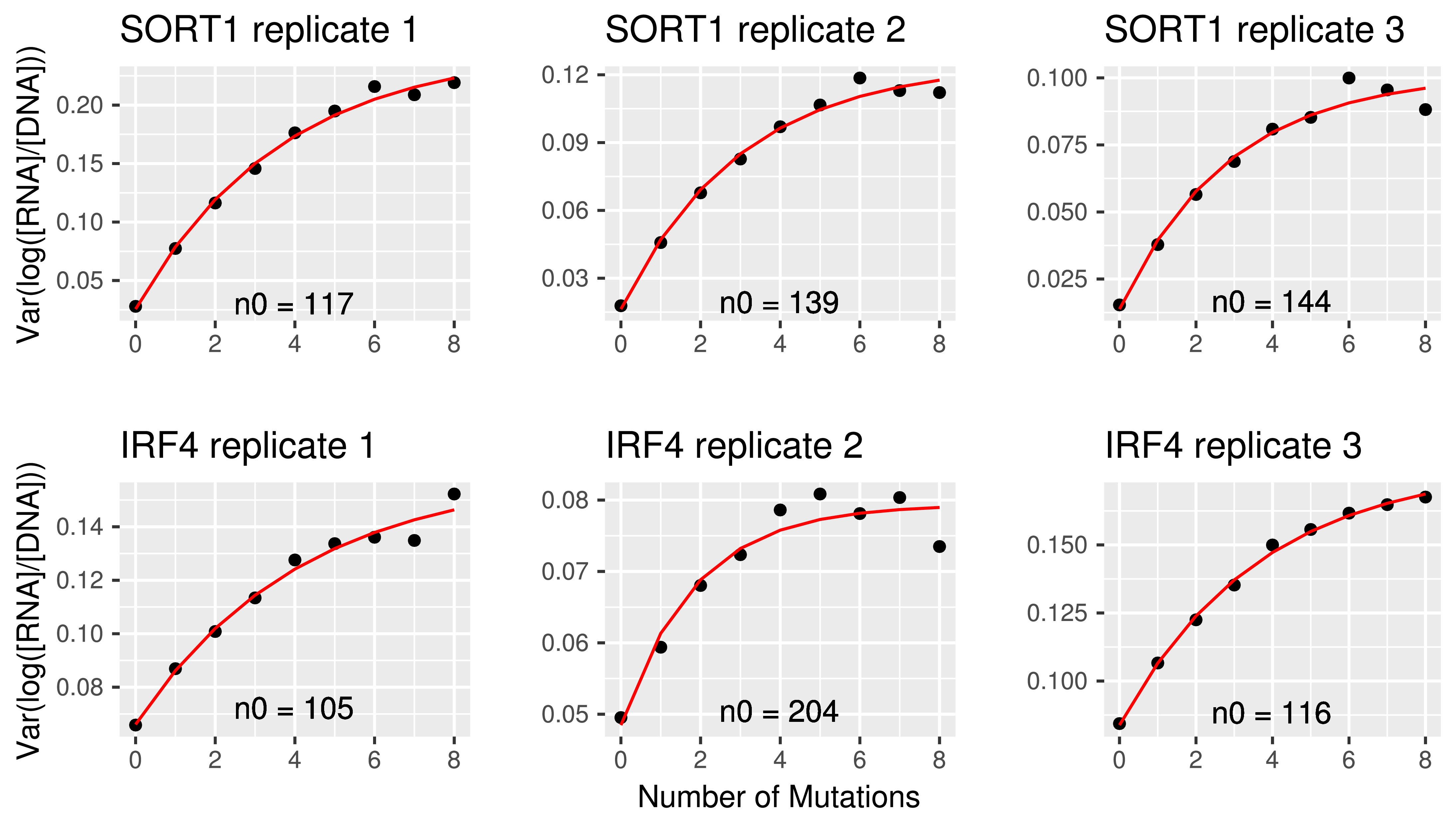

Supplement: S3 Fig — The log variance in expression with respect to the number of mutated nucleotides is reported for each of three experimental replicates for enhancers for the human genes SORT1 and IRF4. The best fit to Eq (3) (red) and the corresponding number of sensitive nucleotides n0 is shown. For details on this analysis see Materials and Methods. (TIF) [file pcbi.1007497.s004.tif]
