# Supplementary material for: An in silico analysis of robust but fragile gene regulation links enhancer length to robustness
Source: PLoS Comput Biol. 2019 Nov 15;15(11):e1007497. doi: 10.1371/journal.pcbi.1007497 (PMC6881076; doi:10.1371/journal.pcbi.1007497)
Supplement: Attachment — Submitted filename: resub_letter.pdf [file pcbi.1007497.s005.pdf]

Laboratory of Pathogen Host Interactions
UMR 5235 Université Montpellier - CNRS

Montpellier, October 8, 2019

Dear Editor,

We have carefully considered the referees comments on our manuscript entitled "An in silico analysis of robust but fragile gene regulation links enhancer length to robustness" by Kenneth Barr, John Reinitz and Ovidiu Radulescu submitted for publication in PLoS Computational Biology as a research article.

We resubmit a revised manuscript that addresses all the specific points made by the referees. This manuscript is submitted in two versions: a corrected version in which the differences with respect to the initial submission are marked in red, and a plain version with no marks. Our answers to the referees are provided as an appendix to this letter.

We hope that the revised version of our manuscript is suitable for publication.

Thank you for your consideration,
Sincerely,

Ovidiu Radulescu, PhD.
Professor, University of Montpellier
On behalf of all the authors

Address : UMR5235-LPHI, Université Montpellier Campus Triolet, cc 107, Bt 24 Place E. Bataillon,
F- 34095 MONTPELLIER Cedex 5 Tél : (33) 4.67.14.92.21

Laboratory of Pathogen Host Interactions
UMR 5235 Université Montpellier - CNRS

Answers to the referees:

Reviewer #1: In this manuscript, Barr, Reinitz and Radulescu dissect the role of mutations, enhancer length and TF concentration on the robustness of gene regulation. They use the classical example of even-skipped gene in Drosophila to show that the enhancers are robust to single nucleotide sequence changes and that this robustness increases with the length of the enhancer. They identified a set of "sensitive" nucleotides that are conserved across several Drosophila species. Finally, the authors show that the transcription rate is highly sensitive to TF concentrations, but this sensitivity is dependent on the A-P axis position. The paper is well written and presents interesting results. The way how definitions are given is really good - first in informal way, then in formal way. The examples makes the technical part easy to understand and easy to read without any prior knowledge of robustness. There are some major points that authors would need to address before I could recommend this paper for publication:

1. The description of the results in the abstract is not so clear. Authors should give a short description how they understand the robustness (as less nucleotides sensitive to mutations, for example).

We have extensively modified the abstract. In the new version the implications of our notion of r-robustness are now briefly defined in plain language. Throughout the abstract, we have made a variety of small changes with the object of stating our main results briefly, specifically, and in plain language.

2. [lines 5-10] Very general description of the robustness in biological systems. It is not clear what actually the problem of the mathematical measures of robustness in biology. Maybe, giving more words on the written examples will give us a better picture.

In lines 4-24 of the new version, we have added several examples of r-robustness: knock-out analysis in yeast, modeling segment polarity patterns of Drosophila and experimental study of multiple mutations of the Drosophila gap gene system. A quantitative analysis of other extant data shows the relevance of r-robustness in the context of regulation of gene expression.

3. [before line 25] There are various examples on robustness, but at the end it makes it more confusing what actually authors are going to investigate. I think, giving more studies related to the technical side of the robustness that authors investigated (TF concentrations and gene expression) would be better.

The examples in lines 18-25 of the new version focus exactly on this point.

4. [after line 25] It feels that it is absolutely different style of writing - very clear descriptions, motivations and problem statements, I really like the «story-telling» style at this part.

Line 25 in the previous version is now line 40. We have addressed the reviewer's point by adding new material in lines 2-5, 9, and 13-24.

5. [line 88] Authors did not formally introduce n_0. It is, not actually obvious. The authors talked before about the insensitive parameters, and should not switch to the sensitive ones without formal introduction.

We provide now a lengthy discussion of n0, as well as a quantitative relation allowing to estimate this number (Eq.4). The definition of n0 is intrinsically related to the "sloppy sensitivity" property of systems with multiscaleness, meaning that some parameters are sensitive and that others are much less. This material can be found at lines 111-114, 116-118, and 122-161.

6. Figure 1C has very few comments in the paragraph. Authors talk about transcriptional repressor but where it is on the plot is not clear.

We have modified Fig 1C to include the role of each TF (Activator, repressor, or both) in the figure. We also added the expression pattern of even-skipped in each pattern to help orient readers. The legend of Fig 1C has been updated to reflect these changes. Additionally, the main text describing this section has been expanded in lines 231-238.

7. In Figure 2, the relationship described by the line on plot 2A is clear only from the figure description, it is not understandable from the paragraph.

We have expanded the text referencing 2A in lines 243-249 and increased its clarity.

8. Figure 3B-E have no comments at all.

Two sentences have been added to reference Figure 3B-E in lines 258-261.

Also, in the results section authors introduced two different robustness measures, but during the reading it is not clear where they talk about the first definition and where about the second one. (It is not critical for understanding at all, but it becomes interesting what is the robustness they are talking about at each particular moment.)

We use multiple measures of robustness throughout the work. ρ is a general measure of robustness, which we use to discuss robustness to changes in TF concentration, while n_0 applies only to r-robustness, which we use to discuss robustness to DNA mutation. We have taken multiple steps make this clear. First, we have updated the first section (Distinguishing types of robustness) to be clarify the meanings of n0 and rho, and to describe when it is appropriate to use n0 instead of rho. Second, we have explicitly mentioned which measure we are using whenever we switch measures within the text.

9. In Figure 5, it would be better to give more comments on different TF concentrations - what exactly is the direction of the relationship in any particular case?

We have added text indicating the direction of the relationship in lines 288-290

10. It would be better to give more comments on figures because the description of the results is not so full in some paragraphs.

More comments on figures is the subject of points 6-9 and 11. In addition to our replies there, we have also expanded the explanation of Fig 4E in lines 269-274.

11. Figure 6C is difficult to understand. The authors need to add better explanations for that figure.

We have greatly expanded the text explaining Fig 6C in lines 299-314. We added a pointer to the appendix, which explains the parameter N in detail on line 303 and the legend to Fig. 6.

12. We previously explored the relationship between TF concentration and observed binding, including for 5 gap TFs at eve-stripped locus, and showed that there is a sensitivity of TF binding to TF concentration (https://academic.oup.com/nar/article/43/1/84/2903035), but this is not generally true for all TFs (https://www.biorxiv.org/content/10.1101/666446v1.abstract). The authors should discuss these results in the context of their findings.

Martin and Zabet 2019 shows that chromatin state, not TF concentration, is responsible for differences in TF binding for some TFs. In our work, chromatin state is set by the TF Zelda, which is uniformly expressed across the embryo, so differences in chromatin cannot explain binding differences across the Drosophila embryo. Still, the Zelda PWM is more predictive of binding than many of the gap gene PWMs (Harrison et al., 2011, *PLoS Genetics*), and Zabet and Adryan (2015, NAR) demonstrate that the inclusion of chromatin state greatly improves the prediction of TF binding at the *even-skipped locus*. Collectively, these experiments highlight the importance of including chromatin state when predicting TF binding with PWMs. Throughout this work, our model of the intact locus includes chromatin state. We do not allow binding within any previously reported closed chromatin regions.

To make it clear that chromatin state is included in our model, we have added a statement on lines 202-203. We would like to thank the reviewer for bringing these important works to our attention. We have included citations to these works on line 203.

Reviewer #2: The manuscript by Barr K.A., Reinitz J. and Radulescu O. "An in silico analysis of robust but fragile gene regulation links enhancer length to robustness" presents a computational study of recently developed and previously published model of Drosophilla embryo patterning relating the nucleotide composition of known enhancer regions
to gene expression of major genes determining the patterning. The authors also exploits previously introduced notions of distributed robustness and r-robustness.

I think this is an interesting study which deserves to appear in PLoS Computational Biology.

I have a couple of important, in my point of view, remarks.

The possibility to connect nucleotide sequences to quantitative properties of phenotypes looks very exciting.

Lphi
Laboratory of Pathogen
Host Interactions
UMR 5235 Université Montpellier - CNRS

However, current manuscript exploits already existing model and already introduced concept.
I think it is necessary to highlight more explicitly the scientific novelty of the study.

The novelty of our results is now stressed at the end of the introduction, in lines 69-71:

"The robustness of the gene regulation model has never been investigated and the previously introduced robustness concepts were only tested on a signalling network model and never in developmental biology.

Later, in lines 75-80, we state:

Finally, we show that expression from longer enhancers is sensitive to changes in fewer nucleotides in both natural and in silico generated enhancers, indicating that enhancer length confers robustness to genetic perturbation. We thus provide a computational proof of the importance of the enhancer length for the robustness of the gene regulation."

Focusing on one particular gene regulation seems to me contradicting to rather general title of the manuscript.
Why it can not be extended other genes known to be involved in patterning?

Our approach is general. Although our findings were validated for the regulation of the gene *eve*, this analysis can be extended to other genes. The reason of our choice is the current availability of data concerning gene regulation from eve enhancers of different lengths and the absence of such data for other enhancers. However, the r-robustness can be readily tested for other enhancers from other organisms. We report this in a new section entitled Human enhancers are r-robust with respect to nucleotide changes at lines 332-343 and in new Supplementary Figure S3.

Otherwise, in the absense of novel experimental data, this seems to be a limitation of the study. Also, the principal conclusion about the character of robustness with respect to mutations (r-robustness) is interesting but it can be rather a consequence of model properties rather than biological reality.I think it would be advantageous to look for the use of independent data to directly or indirectly validate this conclusion.

In this new figure we validate r-robustness using experimental data on human enhancers. Recently Kircher et. al (*Nature Communications* 2019) reported a high-throughput reporter assay on saturation mutagenesis of human enhancers. This scheme incorporates a random number of mutations into enhancers, which is equivalent to our test of r-robustness. For the two enhancers we analysed, variance in expression saturates with increasing r, as predicted by r-robustness. This provides independent experimental validation that r-robustness is neither an artefact of out model, nor is it a unique feature of Drosophila enhancers.

The sequence conservation study used by the authors is an example of using the data independent on the model. However, the results are relatively weak. I would even avoid stating that the results

UMR 5235 Université Montpellier - CNRS

are statistically significant having the p-value just a little below 0.05 threshold.
I think this weakness should be clearly accepted and discussed.

We agree that this p value provides fairly weak statistical support. We have modified the text to reflect this in lines 323-331. In this new material, we adduce reasons why a strong statistical signal might be diluted.

I am also confused by the use of r symbol in the manuscript.
For example, the authors use r to designate the number of perturbed parameters.
At the same time, r participates in the definition of r-robustness,
meaning that r is the maximum number of randomly perturbed parameters,
after which the system loses robustness. Are they the same "r"?

In the new text we distinguish between r in r-robustness (a variable meaning the number of perturbed parameters) and the critical value of r that we denote r* or r1/2 (depending on the criterion for order of magnitude comparison; if a ratio of one half is considered small enough, then r*=r1/2). We hope that this is now clear from our new discussion in the Results subsection “Distinguishing types of robustness” of the relation between the number of sensitive parameters and the critical value of r. This material can be found at lines 146-161.

"This saturating curve is well described by a system with a limited number of sensitive parameters".
It seems that the authors use this sentence to prove the fact of r-robustness.
From what it follows? Can one compare two fits, for r-robustness and distributed
robustness? The major conclusions of the paper are based on this fit, so more formal
approach to prove it is the best fit among some alternative models is needed.

The fact that the curve saturates and thus can be fitted by Eq.3 indicates r-robustness. The alternative distributed robustness predicts a linear dependence between the variance and r, the number of perturbed parameters. This is clearly stated in the Results subsection “Distinguishing types of robustness”. (See in particular lines 162-173)

We have also modified the text describing Fig 2A to make the comparison between distributed and r-robustness clear. This new text appears on lines 243-249.

In order to avoid confusion we have used for all examples the log-variance.

Less important remarks:

The following sentese is confusing to me : "According to this definition, a

Address : UMR5235-LPHI, Université Montpellier Campus Triolet, cc 107, Bt 24 Place E. Bataillon,
F- 34095 MONTPELLIER Cedex 5 Tél : (33) 4.67.14.92.21

property M can be r-robust only for some values of r; typically, it can be robust for small values of r and lose this property for large r."

Can it be at all otherwise (not typically?)

We were referring to the context of r-robustness. Of course, other situations, not covered by the definition of the r-robustness, are possible. In order to avoid any confusion we have reformulated the sentence

"According to this definition, a r-robust property M can have large variance if the number of perturbed parameters is larger than r^*" (lines 102-104).

Here r^* is the critical value of r.

Also, in line 92, the authors suddenly introduce the effect of mutations, without explaining how mutations are connected to model parameters.

To avoid confusion we modified "mutation" to "changes in a sensitive parameter" (lines 116-117).

Does each mutation affect one and only one parameter?

In general, not necessarily. In the studied Drosophila transcription model, the nucleotides in the enhancer sequence are parameters of the model, so each mutation affects one parameter.

It is getting more clear after further reading but it would be better to clearly explain it in the introductory part.

We have added a short sentence:

"In models relating DNA sequence to gene expression each nucleotide in the sequence is a parameter and point mutations act on a single parameter." (lines 158-161)

Formula (4) is supposed to notify "variance of M" while it shows Var(r)

We have changed Var(r) into Var(log(M))

Why for Figure 2 one uses 35.5% embryo length position? If this is just an illustration, then what about other positions, can one get a summary?

Address : UMR5235-LPHI, Université Montpellier Campus Triolet, cc 107, Bt 24 Place E. Bataillon, F- 34095 MONTPELLIER Cedex 5 Tél : (33) 4.67.14.92.21

The summary for other positions is in Figure 2B. Figure 4A shows a detailed fit at 40.5% embryo length.

Minor remarks:

In author summary "sensitive to the levels of regulators" what does it mean? Expression levels?

Concentrations. We now use that word instead of levels.

Line 26: The sentence sounds not clear to me. What represents what and through what?

L26 (now line 40) changed to

In gene networks, the connections between genes represent regulatory interactions

Line 47: "confer robustness to enhancers" sounds unclear. Should be "confer robustness to mutations in enhancers"?

Done (line 61).

Line 51: "it main features" -> "its main features"

Done

Line 73: "distributedly robust" does not sound a good term for me, in terms of language use

Replaced by “is robust in a distributed manner” throughout.

Line 78: I do not quite get the meaning of $\in 2^\{1,...,n\}$ in the expression

2^\{1,...,n\} is the power set of \{1,...,n\}, in other words the set of subsets , the notation is standard.

Line 112: "concentration of measure in high-dimensional spaces, a phenomenon well known in mathematics", a reference would be suitable here,
and not only from Gorban and Radulescu.

New citations now appear on line 29, when measure concentration is first discussed.

Line 135: "contribute robustness", contribute to robustness?

Done

Line 149: "effects of mutations to the environment in trans", what environment? Should be better explained.

We have changed “effects of mutations to the environment in trans” to “effect of ectopic Hb expression” (line 213).

Line 320: "ousting distributed concentration effects." what does it mean?

Excluding (line 435).

Line 380: There is a misprint in the name of the journal

Done (line 527).

Line 526: missing space in the title

Done (line 699)

Line 535: extra comma in the author list

Done (line 708).

Address : UMR5235-LPHI, Université Montpellier Campus Triolet, cc 107, Bt 24 Place E. Bataillon, F- 34095 MONTPELLIER Cedex 5 Tél : (33) 4.67.14.92.21
