# Supplementary material for: An in silico analysis of robust but fragile gene regulation links enhancer length to robustness
Source: PLoS Comput Biol. 2019 Nov 15;15(11):e1007497. doi: 10.1371/journal.pcbi.1007497 (PMC6881076; doi:10.1371/journal.pcbi.1007497)
Supplement: Peer review history

# Peer review: Decision Letter 0

**DOI:** 10.1371/journal.pcbi.1007497.r001

**Authors:** Douglas A Lauffenburger, Ilya Ioshikhes

11 Aug 2019

Dear Dr Radulescu,

Thank you very much for submitting your manuscript 'An in silico analysis of robust but fragile

gene regulation links enhancer length to robustness' for review by PLOS Computational Biology. Your manuscript has been fully evaluated by the PLOS Computational Biology editorial team and in this case also by independent peer reviewers. The reviewers appreciated the attention to an important problem, but raised some substantial concerns about the manuscript as it currently stands. While your manuscript cannot be accepted in its present form, we are willing to consider a revised version in which the issues raised by the reviewers have been adequately addressed. We cannot, of course, promise publication at that time.

Please note while forming your response, if your article is accepted, you may have the opportunity to make the peer review history publicly available. The record will include editor decision letters (with reviews) and your responses to reviewer comments. If eligible, we will contact you to opt in or out.

Your revisions should address the specific points made by each reviewer. Please return the revised version within the next 60 days. If you anticipate any delay in its return, we ask that you let us know the expected resubmission date by email at ploscompbiol@plos.org. Revised manuscripts received beyond 60 days may require evaluation and peer review similar to that applied to newly submitted manuscripts.

In addition, when you are ready to resubmit, please be prepared to provide the following:

(1) A detailed list of your responses to the review comments and the changes you have made in the manuscript. We require a file of this nature before your manuscript is passed back to the editors.

(2) A copy of your manuscript with the changes highlighted (encouraged). We encourage authors, if possible to show clearly where changes have been made to their manuscript e.g. by highlighting text.

(3) A striking still image to accompany your article (optional). If the image is judged to be suitable by the editors, it may be featured on our website and might be chosen as the issue image for that month. These square, high-quality images should be accompanied by a short caption. Please note as well that there should be no copyright restrictions on the use of the image, so that it can be published under the Open-Access license and be subject only to appropriate attribution.

Before you resubmit your manuscript, please consult our Submission Checklist to ensure your manuscript is formatted correctly for PLOS Computational Biology: http://www.ploscompbiol.org/static/checklist.action. Some key points to remember are:

- Figures uploaded separately as TIFF or EPS files (if you wish, your figures may remain in your main manuscript file in addition).

- Supporting Information uploaded as separate files, titled Dataset, Figure, Table, Text, Protocol, Audio, or Video.

- Funding information in the 'Financial Disclosure' box in the online system.

While revising your submission, please upload your figure files to the Preflight Analysis and Conversion Engine (PACE) digital diagnostic tool, https://pacev2.apexcovantage.com PACE helps ensure that figures meet PLOS requirements. To use PACE, you must first register as a user. Then, login and navigate to the UPLOAD tab, where you will find detailed instructions on how to use the tool. If you encounter any issues or have any questions when using PACE, please email us at figures@plos.org.

To enhance the reproducibility of your results, we recommend that you deposit your laboratory protocols in protocols.io, where a protocol can be assigned its own identifier (DOI) such that it can be cited independently in the future. For instructions see here. 

We are sorry that we cannot be more positive about your manuscript at this stage, but if you have any concerns or questions, please do not hesitate to contact us.

Sincerely,

Ilya Ioshikhes

Associate Editor

PLOS Computational Biology

Douglas Lauffenburger

Deputy Editor

PLOS Computational Biology

A link appears below if there are any accompanying review attachments. If you believe any reviews to be missing, please contact ploscompbiol@plos.org immediately:

[LINK]

Reviewer's Responses to Questions

**Comments to the Authors:**

**Please note here if the review is uploaded as an attachment.**

Reviewer #1: In this manuscript, Barr, Reinitz and Radulescu dissect the role of mutations, enhancer length and TF concentration on the robustness of gene regulation. They use the classical example of even-skipped gene in Drosophila to show that the enhancers are robust to single nucleotide sequence changes and that this robustness increases with the length of the enhancer. They identified a set of “sensitive” nucleotides that are conserved across several Drosophila species. Finally, the authors show that the transcription rate is highly sensitive to TF concentrations, but this sensitivity is dependent on the A-P axis position. The paper is well written and presents interesting results. The way how definitions are given is really good - first in informal way, then in formal way. The examples makes the technical part easy to understand and easy to read without any prior knowledge of robustness. There are some major points that authors would need to address before I could recommend this paper for publication:

1. The description of the results in the abstract is not so clear. Authors should give a short description how they understand the robustness (as less nucleotides sensitive to mutations, for example).

2. [lines 5-10] Very general description of the robustness in biological systems. It is not clear what actually the problem of the mathematical measures of robustness in biology. Maybe, giving more words on the written examples will give us a better picture.

3. [before line 25] There are various examples on robustness, but at the end it makes it more confusing what actually authors are going to investigate. I think, giving more studies related to the technical side of the robustness that authors investigated (TF concentrations and gene expression) would be better.

4. [after line 25] It feels that it is absolutely different style of writing - very clear descriptions, motivations and problem statements, I really like the «story-telling» style at this part.

5. [line 88] Authors did not formally introduce n_0. It is, not actually obvious. The authors talked before about the insensitive parameters, and should not switch to the sensitive ones without formal introduction.

6. Figure 1C has very few comments in the paragraph. Authors talk about transcriptional repressor but where it is on the plot is not clear.

7. In Figure 2, the relationship described by the line on plot 2A is clear only from the figure description, it is not understandable from the paragraph.

8. Figure 3B-E have no comments at all. Also, in the results section authors introduced two different robustness measures, but during the reading it is not clear where they talk about the first definition and where about the second one. (It is not critical for understanding at all, but it becomes interesting what is the robustness they are talking about at each particular moment.)

9. In Figure 5, it would be better to give more comments on different TF concentrations - what exactly is the direction of the relationship in any particular case?

10. It would be better to give more comments on figures because the description of the results is not so full in some paragraphs.

11. Figure 6C is difficult to understand. The authors need to add better explanations for that figure.

12. We previously explored the relationship between TF concentration and observed binding, including for 5 gap TFs at eve-stripped locus, and showed that there is a sensitivity of TF binding to TF concentration (https://academic.oup.com/nar/article/43/1/84/2903035), but this is not generally true for all TFs (https://www.biorxiv.org/content/10.1101/666446v1.abstract). The authors should discuss these results in the context of their findings.

Reviewer #2: The manuscript by Barr K.A., Reinitz J. and Radulescu O. "An in silico analysis of robust but fragile gene regulation links enhancer length to robustness" presents a computational study of recently developed and previously published model of Drosophilla embryo patterning relating the nucleotide composition of known enhancer regions

to gene expression of major genes determining the patterning. The authors also exploits previously introduced notions of distributed robustness and r-robustness.

I think this is an interesting study which deserves to appear in PLoS Computational Biology.

I have a couple of important, in my point of view, remarks.

The possibility to connect nucleotide sequences to quantitative properties of phenotypes looks very exciting.

However, current manuscript exploits already existing model and already introduced concept.

I think it is necessary to highlight more explicitly the scientific novelty of the study.

Focusing on one particular gene regulation seems to me contradicting to rather general title of the manuscript.

Why it can not be extended other genes known to be involved in patterning?

Otherwise, in the absense of novel experimental data, this seems to be a limitation of the study.

Also, the principal conclusion about the character of robustness with respect to mutations (r-robustness)

is interesting but it can be rather a consequence of model properties rather than biological reality.

I think it would be advantageous to look for the use of independent data to directly or indirectly

validate this conclusion.

The sequence conservation study used by the authors is an example of using the data independent on

the model. However, the results are relatively weak. I would even avoid stating that the results

are statistically significant having the p-value just a little below 0.05 threshold.

I think this weakness should be clearly accepted and discussed.

I am also confused by the use of r symbol in the manuscript.

For example, the authors use r to designate the number of perturbed parameters.

At the same time, r participates in the definition of r-robustness,

meaning that r is the maximum number of randomly perturbed parameters,

after which the system looses robustness. Are they the same "r"?

"This saturating curve is well described by a system with a limited number of sensitive parameters".

It seems that the authors use this sentence to prove the fact of r-robustness.

From what it follows? Can one compare two fits, for r-robustness and distributed

robustness? The major conclusions of the paper are based on this fit, so more formal

approach to prove it is the best fit among some alternative models is needed.

Less important remarks:

The following sentese is confusing to me : "According to this definition, a

property M can be r-robust only for some values of r; typically, it can be robust for

small values of r and lose this property for large r."

Can it be at all otherwise (not typically?)

Also, in line 92, the authors suddenly introduce the effect of mutations, without

explaining how mutations are connected to model parameters. Does each mutation affect

one and only one parameter? It is getting more clear after further reading but

it would be better to clearly explain it in the introductory part.

Formula (4) is supposed to notify "variance of M" while it shows Var(r)

Why for Figure 2 one uses 35.5% embryo length position? If this is just an illustration,

then what about other positions, can one get a summary?

Minor remarks:

In author summary "sensitive to the levels of regulators" what does it mean? Expression levels?

Line 26: The sentence sounds not clear to me. What represents what and through what?

Line 47: "confer robustness to enhancers" sounds unclear. Should be "confer robustness to mutations in enhancers"?

Line 51: "it main features" -> "its main features"

Line 73: "distributedly robust" does not sound a good term for me, in terms of language use

Line 78: I do not quite get the meaning of $\\in 2^\\{1,...,n\\}$ in the expression

Line 112: "concentration of measure in high-dimensional spaces, a phenomenon well known in mathematics", a reference would be suitable here,

and not only from Gorban and Radulescu.

Line 135: "contribute robustness", contribute to robustness?

Line 149: "effects of mutations to the environment in trans", what environment? Should be better explained.

Line 320: "ousting distributed concentration effects." what does it mean?

Line 380: There is a misprint in the name of the journal

Line 526: missing space in the title

Line 535: extra comma in the author list

**********

**Have all data underlying the figures and results presented in the manuscript been provided?**

Large-scale datasets should be made available via a public repository as described in the *PLOS Computational Biology* data availability policy, and numerical data that underlies graphs or summary statistics should be provided in spreadsheet form as supporting information.

Reviewer #1: Yes

Reviewer #2: Yes

**********

PLOS authors have the option to publish the peer review history of their article (what does this mean?). If published, this will include your full peer review and any attached files.

If you choose “no”, your identity will remain anonymous but your review may still be made public.

**Do you want your identity to be public for this peer review?** For information about this choice, including consent withdrawal, please see our Privacy Policy.

Reviewer #1: Yes: Nicolae Radu Zabet and Liudmila Mikheeva

Reviewer #2: No

# Author comment: Author response to Decision Letter 0

**DOI:** 10.1371/journal.pcbi.1007497.r002

10 Oct 2019

Attachment Submitted filename: resub_letter.pdf Click here for additional data file.

# Peer review: Decision Letter 1

**DOI:** 10.1371/journal.pcbi.1007497.r003

**Authors:** Douglas A Lauffenburger, Ilya Ioshikhes

22 Oct 2019

Dear Dr Radulescu,

We are pleased to inform you that your manuscript 'An in silico analysis of robust but fragile gene regulation links enhancer length to robustness' has been provisionally accepted for publication in PLOS Computational Biology.

Before your manuscript can be formally accepted you will need to complete some formatting changes, which you will receive in a follow up email. Please be aware that it may take several days for you to receive this email; during this time no action is required by you. Once you have received these formatting requests, please note that your manuscript will not be scheduled for publication until you have made the required changes.

In the meantime, please log into Editorial Manager at https://www.editorialmanager.com/pcompbiol/, click the "Update My Information" link at the top of the page, and update your user information to ensure an efficient production and billing process.

One of the goals of PLOS is to make science accessible to educators and the public. PLOS staff issue occasional press releases and make early versions of PLOS Computational Biology articles available to science writers and journalists. PLOS staff also collaborate with Communication and Public Information Offices and would be happy to work with the relevant people at your institution or funding agency. If your institution or funding agency is interested in promoting your findings, please ask them to coordinate their releases with PLOS (contact ploscompbiol@plos.org).

Thank you again for supporting Open Access publishing. We look forward to publishing your paper in PLOS Computational Biology.

Sincerely,

Ilya Ioshikhes

Associate Editor

PLOS Computational Biology

Douglas Lauffenburger

Deputy Editor

PLOS Computational Biology

Reviewer's Responses to Questions

**Comments to the Authors:**

**Please note here if the review is uploaded as an attachment.**

Reviewer #1: The authors addressed all the comments.

Reviewer #2: The authors addressed all my remarks, I think the paper can be accepted now.

**********

**Have all data underlying the figures and results presented in the manuscript been provided?**

Large-scale datasets should be made available via a public repository as described in the *PLOS Computational Biology* data availability policy, and numerical data that underlies graphs or summary statistics should be provided in spreadsheet form as supporting information.

Reviewer #1: Yes

Reviewer #2: Yes

**********

PLOS authors have the option to publish the peer review history of their article (what does this mean?). If published, this will include your full peer review and any attached files.

If you choose “no”, your identity will remain anonymous but your review may still be made public.

**Do you want your identity to be public for this peer review?** For information about this choice, including consent withdrawal, please see our Privacy Policy.

Reviewer #1: Yes: Nicolae Radu Zabet

Reviewer #2: No

# Editor report: Acceptance letter

**DOI:** 10.1371/journal.pcbi.1007497.r004

**Authors:** Douglas A Lauffenburger, Ilya Ioshikhes

7 Nov 2019

PCOMPBIOL-D-19-01037R1 

An in silico analysis of robust but fragile gene regulation links enhancer length to robustness

Dear Dr Radulescu,

I am pleased to inform you that your manuscript has been formally accepted for publication in PLOS Computational Biology. Your manuscript is now with our production department and you will be notified of the publication date in due course.

The corresponding author will soon be receiving a typeset proof for review, to ensure errors have not been introduced during production. Please review the PDF proof of your manuscript carefully, as this is the last chance to correct any errors. Please note that major changes, or those which affect the scientific understanding of the work, will likely cause delays to the publication date of your manuscript. 

Soon after your final files are uploaded, unless you have opted out, the early version of your manuscript will be published online. The date of the early version will be your article's publication date. The final article will be published to the same URL, and all versions of the paper will be accessible to readers.

Thank you again for supporting PLOS Computational Biology and open-access publishing. We are looking forward to publishing your work! 

With kind regards,

Laura Mallard

PLOS Computational Biology | Carlyle House, Carlyle Road, Cambridge CB4 3DN | United Kingdom ploscompbiol@plos.org | Phone +44 (0) 1223-442824 | ploscompbiol.org | @PLOSCompBiol
